# Dysregulated Rbfox2 produces aberrant splicing of Ca_V_1.2 calcium channel in diabetes-induced cardiac hypertrophy

**DOI:** 10.1186/s12933-023-01894-5

**Published:** 2023-07-06

**Authors:** Pengpeng Li, Dongxia Qin, Tiange Chen, Wei Hou, Xinyu Song, Shumin Yin, Miaomiao Song, W.C. Hewith A. Fernando, Xiaojie Chen, Yu Sun, Juejin Wang

**Affiliations:** 1 Key Laboratory of Targeted Intervention of Cardiovascular Disease, Collaborative Innovation Center for Cardiovascular Disease Translational Medicine, Nanjing Medical University, Nanjing, Jiangsu 211166 China; 2 Department of Physiology, Nanjing Medical University, Nanjing, Jiangsu 211166 China

**Keywords:** Alternative splicing, Ca_V_1.2 calcium channel, Diabetes, Cardiomyocyte hypertrophy

## Abstract

**Background:**

L-type Ca^2+^ channel Ca_V_1.2 is essential for cardiomyocyte excitation, contraction and gene transcription in the heart, and abnormal functions of cardiac Ca_V_1.2 channels are presented in diabetic cardiomyopathy. However, the underlying mechanisms are largely unclear. The functions of Ca_V_1.2 channels are subtly modulated by splicing factor-mediated alternative splicing (AS), but whether and how Ca_V_1.2 channels are alternatively spliced in diabetic heart remains unknown.

**Methods:**

Diabetic rat models were established by using high-fat diet in combination with low dose streptozotocin. Cardiac function and morphology were assessed by echocardiography and HE staining, respectively. Isolated neonatal rat ventricular myocytes (NRVMs) were used as a cell-based model. Cardiac Ca_V_1.2 channel functions were measured by whole-cell patch clamp, and intracellular Ca^2+^ concentration was monitored by using Fluo-4 AM.

**Results:**

We find that diabetic rats develop diastolic dysfunction and cardiac hypertrophy accompanied by an increased Ca_V_1.2 channel with alternative exon 9* (Ca_V_1.2_E9*_), but unchanged that with alternative exon 8/8a or exon 33. The splicing factor Rbfox2 expression is also increased in diabetic heart, presumably because of dominate-negative (DN) isoform. Unexpectedly, high glucose cannot induce the aberrant expressions of Ca_V_1.2 exon 9* and Rbfox2. But glycated serum (GS), the mimic of advanced glycation end-products (AGEs), upregulates Ca_V_1.2_E9*_ channels proportion and downregulates Rbfox2 expression in NRVMs. By whole-cell patch clamp, we find GS application hyperpolarizes the current-voltage curve and window currents of cardiac Ca_V_1.2 channels. Moreover, GS treatment raises K^+^-triggered intracellular Ca^2+^ concentration ([Ca^2+^]_i_), enlarges cell surface area of NRVMs and induces hypertrophic genes transcription. Consistently, siRNA-mediated knockdown of Rbfox2 in NRVMs upregulates Ca_V_1.2_E9*_ channel, shifts Ca_V_1.2 window currents to hyperpolarization, increases [Ca^2+^]_i_ and induces cardiomyocyte hypertrophy.

**Conclusions:**

AGEs, not glucose, dysregulates Rbfox2 which thereby increases Ca_V_1.2_E9*_ channels and hyperpolarizes channel window currents. These make the channels open at greater negative potentials and lead to increased [Ca^2+^]_i_ in cardiomyocytes, and finally induce cardiomyocyte hypertrophy in diabetes. Our work elucidates the underlying mechanisms for Ca_V_1.2 channel regulation in diabetic heart, and targeting Rbfox2 to reset the aberrantly spliced Ca_V_1.2 channel might be a promising therapeutic approach in diabetes-induced cardiac hypertrophy.

**Supplementary Information:**

The online version contains supplementary material available at 10.1186/s12933-023-01894-5.

## Background

Diabetes mellitus is characterized by a long-term increased blood glucose level (hyperglycemia) [1], which induces advanced glycation end-products (AGEs) formation as a result of a non-enzymatic interactions in blood [2]. It is well-known that hyperglycemia in diabetes could induce cardiovascular pathological changes, which is closely associated with cardiovascular morbidity and mortality [3]. Beside coronary heart disease and cardiac arrhythmias, the best characterized cardiac complication of diabetes is structural remodeling and impaired function of the heart [4]. Actually, the early feature of diabetic cardiomyopathy (DCM) is impaired diastolic function, and abnormal cardiomyocyte Ca^2+^ handling, which is known to play a key role in the development of cardiac diastolic dysfunction characteristics of early DCM [5], but its underlying mechanisms have yet to be completely clarified.

Physiologically, L-type calcium channel (LTCC) Ca_V_1.2 allows Ca^2+^ ions influx into the cytoplasm of cardiomyocytes in response to membrane depolarization [6]. This process leads to a transient increase in the intracellular Ca^2+^ concentration by calcium-induced calcium release (CICR) that induces gene transcriptions, named as excitation-transcription coupling, to maintain proper structures and functions of heart [7]. If the intracellular calcium homeostasis is disturbed, the cardiac structures and functions will be dysregulated, which takes an important role in DCM [8, 9]. In DCM, the function of Ca_V_1.2 channels is also found to be changed within the cardiomyocyte, probably affecting their membrane expression [10]. However, the detailed mechanism(s) for impaired calcium handling of cardiomyocytes under diabetic hyperglycemia is largely unclear.

Ca_V_1.2 calcium channel is composed of 4 subunits (α_1C_, β, α_2_δ and γ) [11], among them, the pore-forming α_1C_ is the main subunit, which conducts Ca^2+^ ions from extracellular into intracellular. Human α_1C_ subunit is encoded by *CACNA1C* gene with 55 exons, of which at least 19 exons are subjected to alternatively spliced [12], these spliced exons are known to alter the physiological and pharmacological functions of Ca_V_1.2 channel [13–16]. Under pathological conditions, alternative splicing (AS) events of Ca_V_1.2 channel are found to be aberrant in cardiovascular tissues [17]. For example, Ca_V_1.2 alternative exon 9*, located in the I-II loop of α_1C_ subunit, was increased in chronic myocardial infraction [18] and hypertrophic heart [19]. Inclusion of alternative exon 9* changed the electrophysiological and pharmacological functions of Ca_V_1.2 channels [20, 21]. Moreover, galectin-1, a carbohydrate-binding protein, is found to bind to Ca_V_1.2 channel in an exon 9*-specific manner [15], which subtly affects channel functions in cardiomyocyte [19] and vascular smooth muscle [15, 22]. But in the diabetic heart, the change of Ca_V_1.2 AS events and its regulation mechanism(s) remains elusive.

AS events are modulated by the splicing factor, an RNA-binding protein. To date, multiply splicing factors could modulate Ca_V_1.2 AS events [17]. For Ca_V_1.2 alternative exon 9*, there are 2 kinds of splicing factors, RBM20 [23] and Rbfox [24], which are known to regulate its AS events. Mechanistically, RBM20 bound to introns surrounding exon 9*, promoted the inclusion of exon 9* in Ca_V_1.2 channel [23]. Whereas, Rbfox proteins bound to UGCAUG elements of *Cacna1c* pre-mRNAs to repress Ca_V_1.2 alternative exon 9* in neural development [24] and vascular smooth muscle [16, 25]. Previously, we discovered that Rbfox2 regulates the expression of Ca_V_1.2 alternative exon 9* in vascular smooth muscles by taking a key role in the pathogenesis of hypertension [16]. Recent studies indicated that dysregulation of Rbfox2 is associated with heart development [26], heart failure [27], and cardiac arrhythmias in myotonic dystrophy [28]. Moreover, dysregulation of Rbfox2 disrupted AS patterns of multiply genes, which was thought to be an early event in DCM [29]. However, the roles of Rbfox2 in the regulation of cardiac Ca_V_1.2 AS and channel function remained unidentified in diabetic heart.

In this study, we brought to light that AGEs, not glucose, play a key role in the modulation of cardiac Ca_V_1.2 channels by regulating Rbfox2-mediated AS events in cardiomyocytes under diabetic hyperglycemia. In detail, AGEs-induced dysregulation of Rbfox2 yielded aberrant splicing of Ca_V_1.2 channel, which hyperpolarized the window currents and increased intracellular calcium concentration ([Ca^2+^]_i_), and finally led to cardiac hypertrophy in diabetes.

## Methods

### Animal model and cell line

All animal works have been approved by and performed in accordance with the Institutional Animal Care and Use Committee of Nanjing Medical University and conformed to the Guide for the Care and Use of Laboratory Animals (NIH publication, 8th edition, 2011). Adult male Sprague-Dawley (SD) rats were fed with standard diet or high-fat diet (HFD) and tap water ad libitum at constant ambient temperature (25 °C) and humidity. Diabetic rat model was established by intraperitoneal injection of streptozotocin (STZ, 25 mg/kg in 10 mmol/L citrate buffer at pH = 4.6) for 5 consecutive days and fed with HFD. Control rats received the same volume of citrate buffer (0.1 mol/L Na citrate, pH = 4.5) and fed with standard diet. Blood glucose levels were measured using a glucometer (ACCU-CHEK, Roche, USA), and the body weight of the rats were measured every 2 weeks. For tissue collection, the rats were euthanized by overdose CO_2_, then the ventricles were harvested for further experiments, cell surface area (CSA) of cardiomyocytes obtained from different rats’ heart tissue was calculated according to the images of HE staining. The levels of AGEs in blood serum and the lysate of cardiac tissue were measured by using an AGEs ELISA kit (Jiancheng Bioengineering Institute, Nanjing, China). H9c2 rat cardiac myoblast cells were obtained from American Type Culture Collection (Rockville, MD, USA).

### Echocardiography

Transthoracic echocardiographic measurements were performed as described in the previous study [19] with Vevo 2100 ultrasound set-up (Visual Sonics, Canada). Rats were anesthetized with isoflurane (2% with O_2_, 1 L/min). Left ventricle wall thickness and chamber dimensions were determined in the parasternal short-axis view (M-mode), from which measures of left ventricle ejection fraction (LVEF) and fractional shortening (FS) were derived. 2D images of the heart were obtained from the 4-chamber apical view to assess mitral blood inflow and tissue Doppler velocities. All measurements were averaged over 6 successive cardiac cycles and performed by an echocardiographer who was blinded throughout the whole experiment.

### Glycated serum preparation

For preparation of glycated serum (GS), dialyzed-fetal bovine serum (FBS) was incubated under sterile conditions with D-glucose (9 g/mL) at 37 °C for 3 weeks [30, 31]. The serum was then extensively dialyzed against 0.1 mol/L PBS, pH 7.4, to remove unincorporated glucose within the serum. The non-glycated serum (NG) was also incubated under the same conditions, but without D-glucose. The concentrations of AGEs in GS were determined by ELISA assay, which was 96.12 ± 8.85 ng/mL.

### RNA extraction and RT-PCR

The total RNA was extracted from cells or tissues by using Trizol reagent (Invitrogen), following the manufacturer’s instructions, and then 1 µg of total RNA was reverse-transcribed to cDNA using HiScript III QRT Super-Mix reverse transcriptase (Vazyme, China). RT-PCR was carried out using Rapid Taq Master Mix (Vazyme). 2% agarose gel was used to separate the PCR products. Quantitative real-time PCR (qPCR) was performed on Step-One Plus real-time PCR System (Applied Biosystems, Thermo Fisher Scientific, USA). Reactions were performed in 96-well plates with 20 µL volume including 100 ng cDNA, 200 nmol/L of each primer and 10 µL AceQ qPCR SYBR Green Master Mix (Vazyme). *Actb* mRNA was used to normalize the expression of target genes. The 2^−ΔΔCT^ method was used to analyze the data. Sequences of the primers are listed in Table S1.

### Primary neonatal rat ventricular cardiomyocytes isolation and cell treatment

NRVMs were extracted from SD rats of 1 to 2-days old by using Pierce Primary Cardiomyocyte Isolation Kit (Cat No. 88281, Thermo Fisher Scientific), which contains enzyme #1 (with papain) and enzyme #2 (with thermolysin). Neonatal hearts were placed into separate 1.5 mL centrifuge tubes with 300 µL HBSS. Cardiac tissues were then cut into small pieces with a curved scissors inside a sterile centrifuge tube. The minced tissue was then digested by adding 0.2 mL of enzyme #1 and 10 µL enzyme #2. Next, the tubes were incubated in a shaker under 37 °C for 30–35 min. The mixture was then transferred into another dish with DMEM medium (Gibco) containing 10% FBS (Gibco) and 1% penicillin/streptomycin (Sigma). NRVMs or H9c2 cells were transfected with siRNAs targeting Rbfox2 mRNA (Table S2) with Lipofectamine 3000 reagent (Invitrogen) according to the manufacturer`s instruction. The cells treated with AGEs (bs-1158P, Bioss, Beijing, China) or methylglyoxal (MGO) (MedChemExpress, Shanghai, China) were collected for further analysis.

### Extraction of membrane protein

Membrane protein extraction was performed on ice. The heart tissue or cells were homogenized in the lysis buffer (KPG350, KeyGEN, China) added with protease inhibitor and dithiothreitol. Then vortex mixing was done for 30 s and place on the ice for 1 min, repeated for 5 cycles. The lysate was centrifuged at 12,000 rpm (MicroCL 17R, Thermo Fisher Scientific) for 10 min. The pellets were collected and homogenized in an extraction buffer solution. Then vortex mixed for 30 s, and place on the ice for 5 min, 5 cycles. The lysate was centrifuged at 12,000 rpm for 10 min again, and the supernatant was collected as the membrane protein.

### Western blotting

Total protein was extracted from cells or left ventricular tissues. The whole operation was carried out on ice. The collected cells or the ground tissues were lysed with RIPA lysis buffer, which added protease inhibitors. Lysates were centrifuged for 15 min at 12,000 rpm under 4℃. The supernatants were used for Western blotting after determining its protein concentration by Bradford assay (Yeasen, China). The protein was separated on 10% SDS-PAGE and transferred to PVDF membranes. After blocking, PVDF membranes were incubated with different primary antibodies overnight at 4℃ or 2 h at 25℃.

Primary antibodies against targeted proteins were used: β-actin (1 mg/mL, HRP-66,009, Proteintech), Rbfox2 (1 mg/mL, NB110-40588, rabbit polyclonal, Novus), Ca_V_1.2 α_1C_ (1.6 µg/mL, ACC-003, rabbit polyclonal, Alomone), Na-K ATPase (1.0 µg/mL, Ab7671, mouse monoclonal, Abcam), RBM20 (0.5 mg/mL, NBP2-27509, goat polyclonal, Novus). The secondary antibodies used were HRP-conjugated goat anti-mouse (0.02 µg/mL, SA00001-1, Proteintech) or anti-rabbit IgG (0.02 µg/mL, SA00001-2, Proteintech), or rabbit anti-goat IgG (A21030, Abbkine). We used an imaging system (Tanon 5200, China) to visualize the blot with the enhanced chemiluminescence reagent (Pierce, Thermo Fisher Scientific).

### Immunofluorescence staining

CSA of isolated NRVMs was determined by immunofluorescence staining. Briefly, the NRVMs were fixed in 4% paraformaldehyde for 15 min, permeabilized with 0.1% Triton X-100 in PBS for 5 min, then blocked with 0.3% BSA in PBS for 40 min at 25℃ and incubated with primary antibody α-actinin (1 mg/mL, 66895-1-lg, mouse monoclonal, Proteintech) overnight at 4℃. After washing with PBS 3 times for 5 min, the NRVMs were incubated with secondary antibody labeled with Alexa Fluor 488 (R37114, Molecular Probes) for 1 h at 25℃. Then, DAPI was used to label the nuclei. The fluorescence signaling was captured by a confocal laser scanning microscope (LSM710, Carl Zeiss, German). Image J software was used to calculate surface area of the cells.

### Measurement of intracellular Ca^2+^ concentration

[Ca^2+^]_i_ was measured using Fluo-4 AM (Molecular Probes, Thermo Fisher Scientific, USA) as previously described [19]. Briefly, NRVMs were put on the dishes loaded with Fluo-4 AM (3 µmol/L reconstituted in DMSO) and 0.1% Pluronic™ F-127 (P3000MP, Thermo Fisher Scientific, USA) in Hanks’ balanced salt solution. This operation should be performed as quickly as possible to avoid decomposition with subsequent loss of cell loading capacity. Images were obtained by time series scanning mode and sampled at 1 fps by using a confocal laser scanning microscope (LSM710, Carl Zeiss, German), and the fluorescence intensity was analyzed offline. To standardize the fluorescence intensity of intracellular Ca^2+^ indicators, the change of [Ca^2+^]_i_ was calculated by the equation: Δ[Ca^2+^]_i_ = Δ*F*/*F*_0_ = (*F* − *F*_0_)/*F*_0_, where *F* is the fluorescence intensity of NRVMs at any given time and *F*_*0*_ is the basal fluorescence intensity prior to an experimental manipulation.

### Whole-cell recording of calcium channel currents

The bath solution for recording NRVMs contained (in mmol/L) 132 TEA-Cl, 10 HEPES, 1 MgCl_2_, 10 BaCl_2_, 10 glucose, pH was adjusted to 7.4, osmolarity was 300–310 mOsm. The internal solution contained (in mmol/L) 130 CsCl, 5 EGTA, 1 MgCl_2_, 10 HEPES, 2 Na_2_ATP, 0.5 GTP, 10 glucose, pH was adjusted to 7.2, osmolarity was 290–300 mOsm. Whole-cell currents obtained under a voltage-clamp with an Axopatch 200B amplifier (Molecular Device, San Jose, California, USA), were filtered at 1–5 kHz and sampled at 5–50 kHz, and the series resistance was typically < 5 MΩ after > 70% compensation. The P/4 protocol was used to subtract the leak and capacitive transients online.

Ca_V_1.2 channel currents were recorded by holding the cell at -55 mV before stepping to various potentials from − 50 to 50 mV over 1500 ms, then the currents were recorded by a 200 ms test pulse at 0 mV. Then the current-voltage (*I–V*) relationship curve was fitted to the equation: *I* = *G*_max_ (*V* − *E*_rev_) / (1 + exp([*V* – *V*_0.5_]/*k*), where *G*_max_ is the maximum conductance, *V* is the testing potential, *E*_rev_ is the reversal potential, *V*_0.5_ is half-activation potential, and *k* is slope rate. Steady-state activation (SSA) curve was derived from *I*–*V* protocol, and calculated by the equation of *G* = *I* / (*V* – *E*_rev_), where *G* is conductance, and the curve was fitted with the Boltzmann equation [32]. The steady-state inactivation (SSI) curves were obtained from each test pulse which normalized to the maximal current amplitude of the normalizing pulse. The *SSI* curve was fitted with a single Boltzmann equation: *I*_relative_ = *I*_min_+(*I*_max_−*I*_min_)/(1 + exp((*V*_0.5,inact_−*V*)/*k*), where *I*_relative_ is the normalized current; *V*_0.5,inact_ is the potential for half-inactivation, and *k* is the slope value.

### Statistical analysis

Data are presented as the mean ± standard error of mean (S.E.M.). n number refers to biological repeats. The statistical significance was analyzed using a Student’s unpaired *t*-test, one-way or two-way ANOVA followed by post hoc multiple comparisons. A value of *P* < 0.05 was set as the significant statistical difference.

## Results

### The heart from diabetic rat model shows hypertrophic morphology and impaired diastolic function

**Fig. 1:**
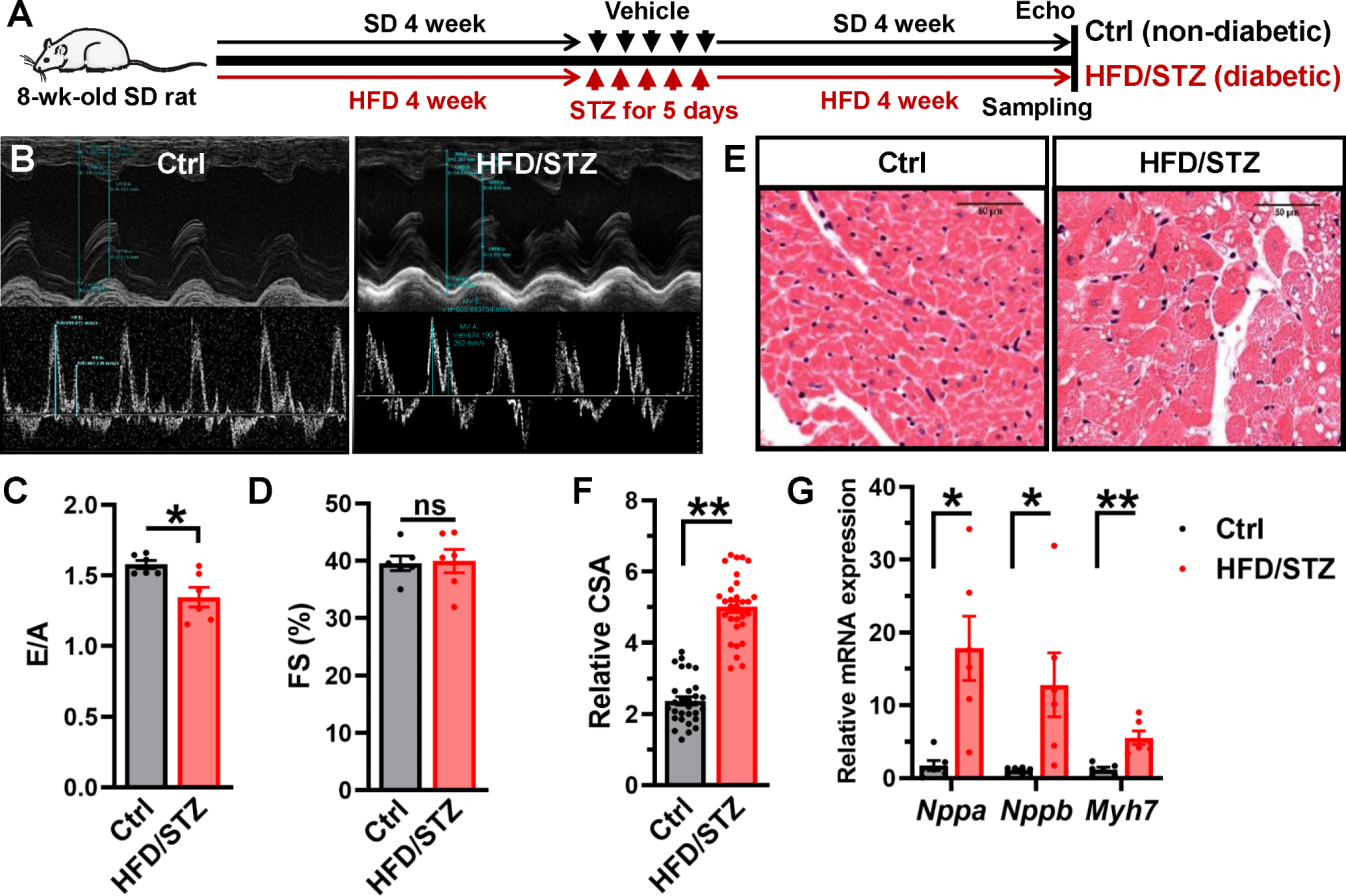
**Diabetic hearts show impaired diastolic function and hypertrophic phenotype**. (**A**) Diabetic rat models were established by using the combination of high-fat diet (HFD) and streptozotocin (STZ) (i.p. injection). Control rats received the same volume of vehicle (citrate buffer) and fed with standard diets. After 8 weeks, the cardiac functions were measured by echocardiography and hearts were collected for further investigations. (**B**) Representative M-mode traces and left ventricle (LV) Doppler images in control and HFD/STZ-treated rats. (**C**) E/A ratio (ratio of early left ventricular diastolic flow velocity and late left ventricular diastolic flow velocity) was analyzed and presented as a bar chart. n = 6 rats for each group. *P = 0.0121, unpaired *t* test. (**D**) Fractional shortening (FS) was also analyzed. n = 6 rats for each group. *P* = 0.8752, unpaired *t* test. ns indicates no significant differences. (**E**) Representative images of HE staining for cardiac tissue from control and HFD/STZ-treated rats. (**F**) Cardiomyocyte cell surface area (CSA) was analyzed and showed as a bar chart. n = 30 cells from 3 rats for each group. **P < 0.0001, unpaired *t* test. (**G**) Rat *Nppa*, *Nppb* and *Myh7* mRNAs were determined by real-time RT-PCR in the rats, and rat *Actb* mRNA was measured as internal control. n = 6 rats for each group. *P < 0.05, **P < 0.01, unpaired *t* test with Welch’s correction

Diabetic rat model was established by HFD in combination with STZ (Fig. 1A). Five days post administration with STZ, the rats showed an increase in blood glucose (hyperglycemia) with decreased body weight (Figure S1A-B). Furthermore, heart weight/body weight ratio of HFD/STZ-treated rats was increased when compared with control rats (Table S3). Intraperitoneal glucose tolerance test showed that HFD/STZ-treated rats had an impaired glucose tolerance (Figure S1C), confirming the presence of diabetic phenotype. Echocardiographic assessment found that LV diastolic function of HFD/STZ-treated rats was significantly decreased in comparison with the control rats (Fig. 1B), as indicated by a lower ratio between early diastolic mitral flow velocity (E) and late diastolic mitral flow velocity (A) (Fig. 1C, Table S4). But the LV systolic function was preserved (Fig. 1D, Table S4), which is similar to patients with an early stage of DCM. Cardiac hypertrophy is one of common characteristics in diabetic cardiac dysfunction. We performed HE staining on heart sections to determine cardiomyocyte sizes and measured cardiomyocyte CSA of the hearts (Fig. 1E), upon obtaining the results we observed that the hearts from HFD/STZ-treated rats have increased cardiomyocyte surface area in comparison with control rats (Fig. 1F), indicating the phenotype of cardiac hypertrophy. This was further evident by higher mRNA expressions of hypertrophic genes transcriptions, including *Nppa*, *Nppb* and *Myh7* in HFD/STZ-treated rats cardiac tissues compared with controls (Fig. 1G). These data identified the diabetic hypertrophic phenotypes in the heart from HFD/STZ-treated rats.

### Aberrant splicing of Ca_V_1.2 channel in diabetic heart

**Fig. 2:**
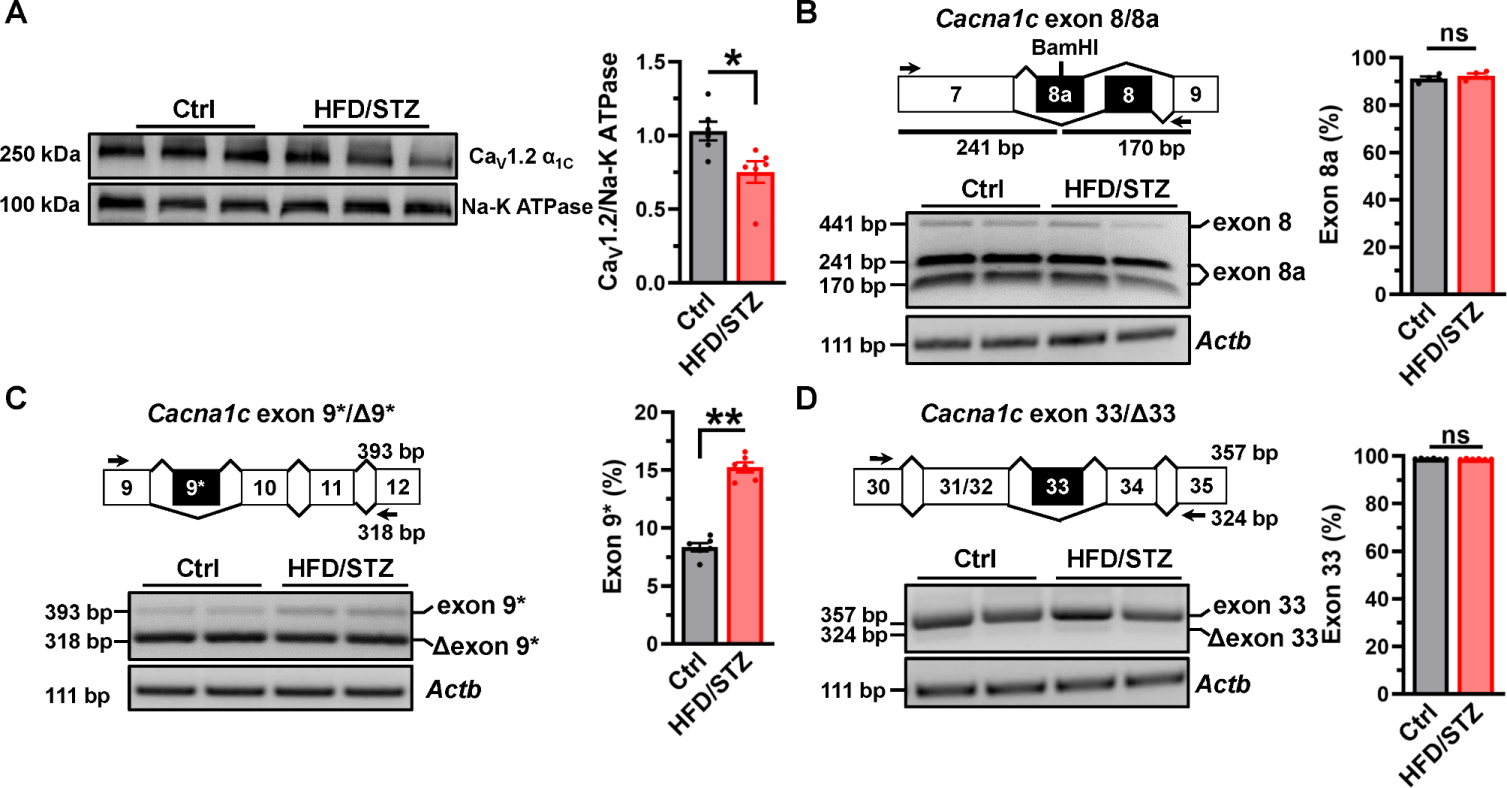
**Ca**_**V**_1.2 alternative exon 9* is specifically increased in diabetic heart. (**A**) The membrane expression of Ca_V_1.2 α_1C_ was detected by Western blotting in heart tissues from control and HFD/STZ-treated rats, Na-K ATPase protein was detected as internal control. The relative band densities were analyzed and normalized to Na-K ATPase. n = 6 rats for each group. *P = 0.0166, unpaired *t* test. (**B**) Schematic diagram shows the locations of the PCR primers designed to amplify and detect rat Ca_V_1.2 inclusive of or in the absence of alternative exons in cardiac tissues. Total RNA was extracted from hearts, and PCR products amplified from cDNA libraries were separated on 2.5% agarose gel. *Actb* mRNA was detected as internal control. Rat *Cacna1c* mRNAs with exon 8 or 8a were amplified by RT-PCR, followed by digestion with restriction endonuclease BamHI. The value for percent exon 8a inclusion were the lower 2 bands’ intensity divided by the sum of the intensities of upper and lower bands. n = 4 rats for each group. *P* = 0.4107, unpaired *t* test. (**C**) The value for percent exon 9* inclusion was the upper band intensity divided by the summed intensities of upper and lower bands. n = 6 rats for each group. **P < 0.0001, unpaired *t* test. (**D**) The value for percent exon 33 inclusion was also presented as a bar chart. n = 6 rats for each group. *P* = 0.4107, unpaired *t* test. ns indicates no significant differences

Ca_V_1.2 calcium channel is the key path for the calcium influx in cardiomyocyte, which thereby triggers the CICR [33]. We found that the membrane expression of Ca_V_1.2 channels was decreased in cardiac tissues from HFD/STZ-treated rats (Fig. 2A), indicating Ca_V_1.2 channels on cell membrane of the heart are diminished under diabetic hyperglycemia, possibly attributed by a repaired trafficking mechanism [10]. Ca_V_1.2 AS events have been known to be changed in cardiovascular diseases, which therefore modulate the functions of Ca_V_1.2 channels [13, 14, 16, 19]. Next, we explored the Ca_V_1.2 AS events in the diabetic hearts. The percentage of Ca_V_1.2 channel with exon 8 or exon 8a was measured through RT-PCR followed by restriction endonuclease BamHI digestion. The results showed that there were no significant differences in Ca_V_1.2 channels with exon 8a between the cardiac tissue of HFD/STZ-treated rats and controls (Fig. 2B). Then, specific primers were used to amplify and detect the Ca_V_1.2 inclusive of exon 9* or exon 33 in cardiac tissue. In comparison to the controls, the proportion of Ca_V_1.2 with alternative exon 9* inclusion (Ca_V_1.2_E9*_) was increased by ~ 7% in HFD/STZ-treated rat hearts (Fig. 2C), but the proportion of Ca_V_1.2 channels with exon 33 was unexpectedly unchanged (Fig. 2D). These observations indicated the specific splicing event of Ca_V_1.2 channels, alternative exon 9*, is increased in the hearts of diabetic rats.

### Expression of Rbfox2 is decreased in diabetic heart

**Fig. 3:**
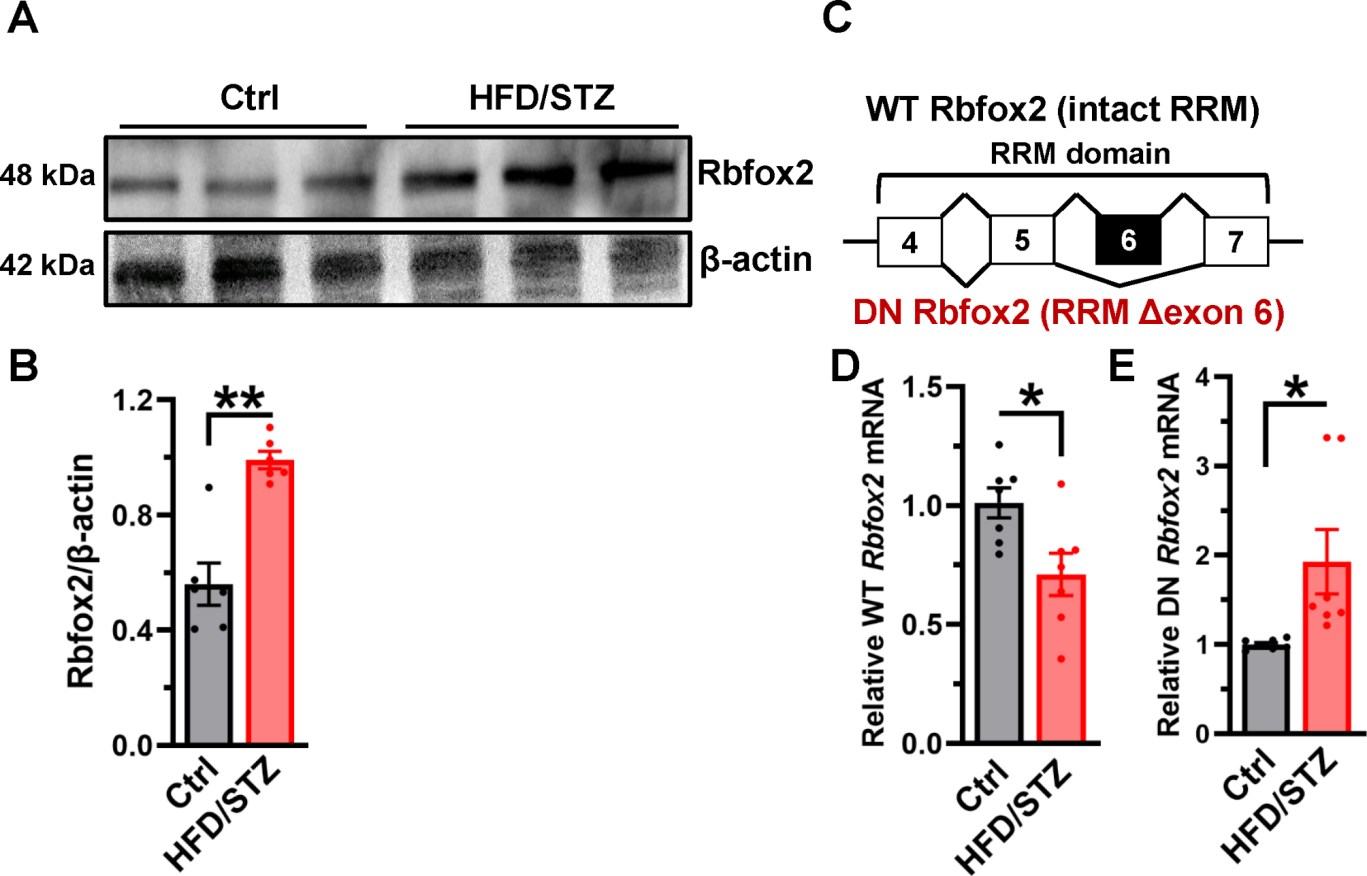
**Expression of Rbfox2 is dysregulated in the hearts from diabetic rats**. (**A**) The expression of Rbfox2 in the rat hearts was detected by Western blotting; β-actin was detected as loading control. (**B**) Relative expression of Rbfox2 was normalized to β-actin. n = 6 rats for each group. **P = 0.0003, unpaired *t* test. (**C**) Different from wild-type (WT) Rbfox2, dominant-negative (DN) Rbfox2 is generated by exclusion of exon 6, which encodes half of the RNA recognition motif (RRM). (**D**) Using specific primers, we determined the WT *Rbfox2* mRNA levels of the hearts by real-time RT-PCR; rat *Actb* mRNA was measured as internal control. n = 7 rats for each group. *P = 0.0171, unpaired *t* test. (**E**) DN *Rbfox2* mRNA was also checked by real-time RT-PCR. n = 7 rats for each group. *P = 0.0423, unpaired *t* test with Welch’s correction

Ca_V_1.2 alternative exon 9* is known to be regulated by splicing factors RBM20 [23] and Rbfox proteins [16, 25]. However, the expression of RBM20 in HFD/STZ-treated hearts remained unchanged (Figure S2), implying that RBM20 may not involve in the AS regulation in diabetic heart. Next, we checked the expression of Rbfox2 proteins in the heart, and clearly observed a significant increase in the expression of Rbfox2 in diabetic hearts compared with the controls (Fig. 3A-B). As Rbfox2 exists 2 isoforms, wild-type (WT) Rbfox2 has a complete RNA-binding domain, and the other form excludes exon 6 and only encodes half of the RNA recognition motif hence forming dominant-negative (DN) Rbfox2 (Fig. 3C) [34]. In order to illuminate the dysregulated Rbfox2, we examined the mRNA levels of WT and DN Rbfox2 in diabetic cardiac tissues. It was found that in comparison with control rats, the level of WT *Rbfox2* mRNA was decreased in the heart from HFD/STZ-treated rats (Fig. 3D). However, the mRNA level of DN *Rbfox2* was markedly increased (Fig. 3E). These results suggest that the upregulated Rbfox2 proteins found in heart from diabetic rats is presumably attributed to increased expression of DN isoform.

### Rbfox2 directly regulates AS of Ca_V_1.2 channel in cardiomyocytes

**Fig. 4:**
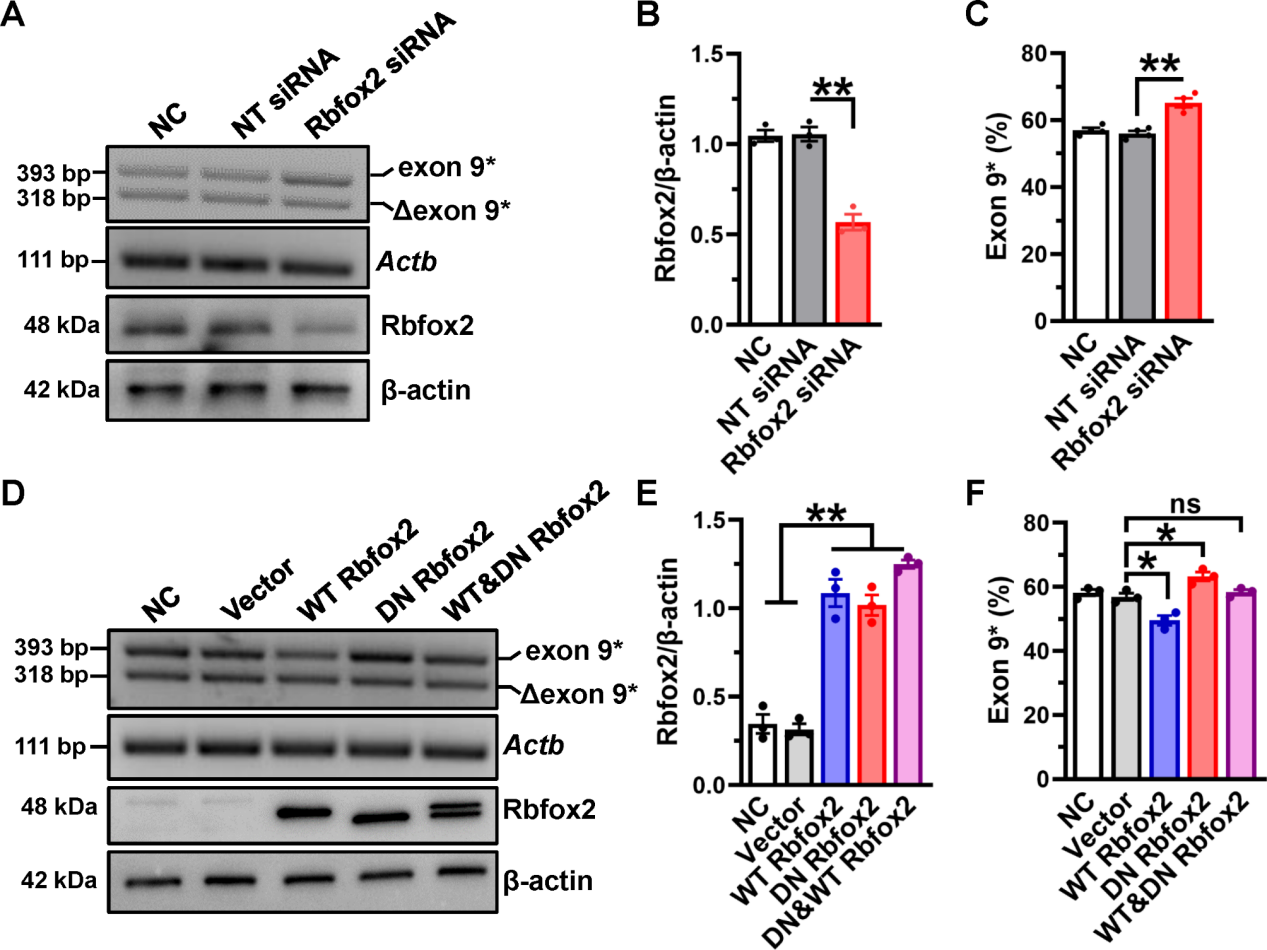
**Rbfox2 specifically modulates Ca**_**V**_1.2 alternative exon 9* splicing in cardiomyocyte. (**A**) H9c2 cells were transfected with nontargeting (NT) or Rbfox2 siRNA for 48 h. The endogenous expression of Rbfox2 protein was detected by Western blotting, the β-actin was detected as internal control. PCR product of Ca_V_1.2_E9*_ channel was amplified from cDNA libraries and separated on 2.5% agarose gel. *Actb* mRNA was detected as internal control. (**B**) The relative expression of Rbfox2 was normalized to β-actin. (**C**) The value for percent Ca_V_1.2_E9*_ inclusion was the upper band intensity divided by the summed intensities of upper and lower bands, and presented as a bar chart. (**D**) H9c2 cells were transfected with an empty vector, WT Rbfox2, DN Rbfox2 or WT plus DN Rbfox2 expression plasmids, nontreated cells were set as negative control (NC). After 48 h incubation, the expression of Rbfox2 protein was detected by Western blotting, the β-actin was detected as internal control. PCR products were separated on 2–3% agarose gel, which was used to check the proportions of Ca_V_1.2_E9*_ channels. (**E**) Relative expression of Rbfox2 was normalized to β-actin. (**F**) The proportion of Ca_V_1.2_E9*_ channels were analyzed and presented by a bar chart. n = 3 independent experiments. *P < 0.05, **P < 0.01, one-way ANOVA followed by a Tukey’s post hoc test

Rbfox2 regulates Ca_V_1.2 AS events in neuronal development [24] as well as vascular smooth muscle [16]. In order to investigate the effects of Rbfox2 on Ca_V_1.2 AS in cardiomyocyte, siRNA transfections efficiently knocked down Rbfox2 expression in H9c2 cells (Fig. 4A-B). Furthermore, specific primers were applied to detect the proportion of Ca_V_1.2_E9*_ of H9c2 cells, and mentioned that it increased ~ 8% after knocking down Rbfox2 (Fig. 4A and C). We also used siRNA approaches to knockdown Rbfox2 in NRVMs, the results also showed that the proportion of Ca_V_1.2_E9*_ channels is obviously increased (Figure S3A-C), these data were consistent with previous findings in VSMCs [16]. However, we didn’t find that Rbfox2 knockdown could change the proportion of Ca_V_1.2_E33_ channels in NRVMs (Figure S4A-B).

In contrast, we analyzed the effects of overexpression of Rbfox2 on the Ca_V_1.2_E9*_ proportion of cardiomyocyte, and found that WT Rbfox2 overexpression decreases the proportion of Ca_V_1.2_E9*_ channels. Notably, co-expression with equal amounts of WT and DN Rbfox2 failed to change the proportion of Ca_V_1.2_E9*_ channels in H9c2 cells (Fig. 4D-F). Furthermore, increasing the expression of DN isoform of Rbfox2 raised the proportion of Ca_V_1.2_E9*_ channels in a dose-dependent manner (Figure S3D-F). Together, our results showed that the AS of Ca_V_1.2 channel can be dynamically regulated by Rbfox2 in cardiomyocytes, thus aberrant splicing of Ca_V_1.2 is indeed attributed to dysregulated Rbfox2 in diabetes-induced cardiomyopathy.

### Glycated-serum (GS), not glucose, induces Rbfox2 downregulation and Ca_V_1.2 aberrant splicing in cardiomyocyte

**Fig. 5:**
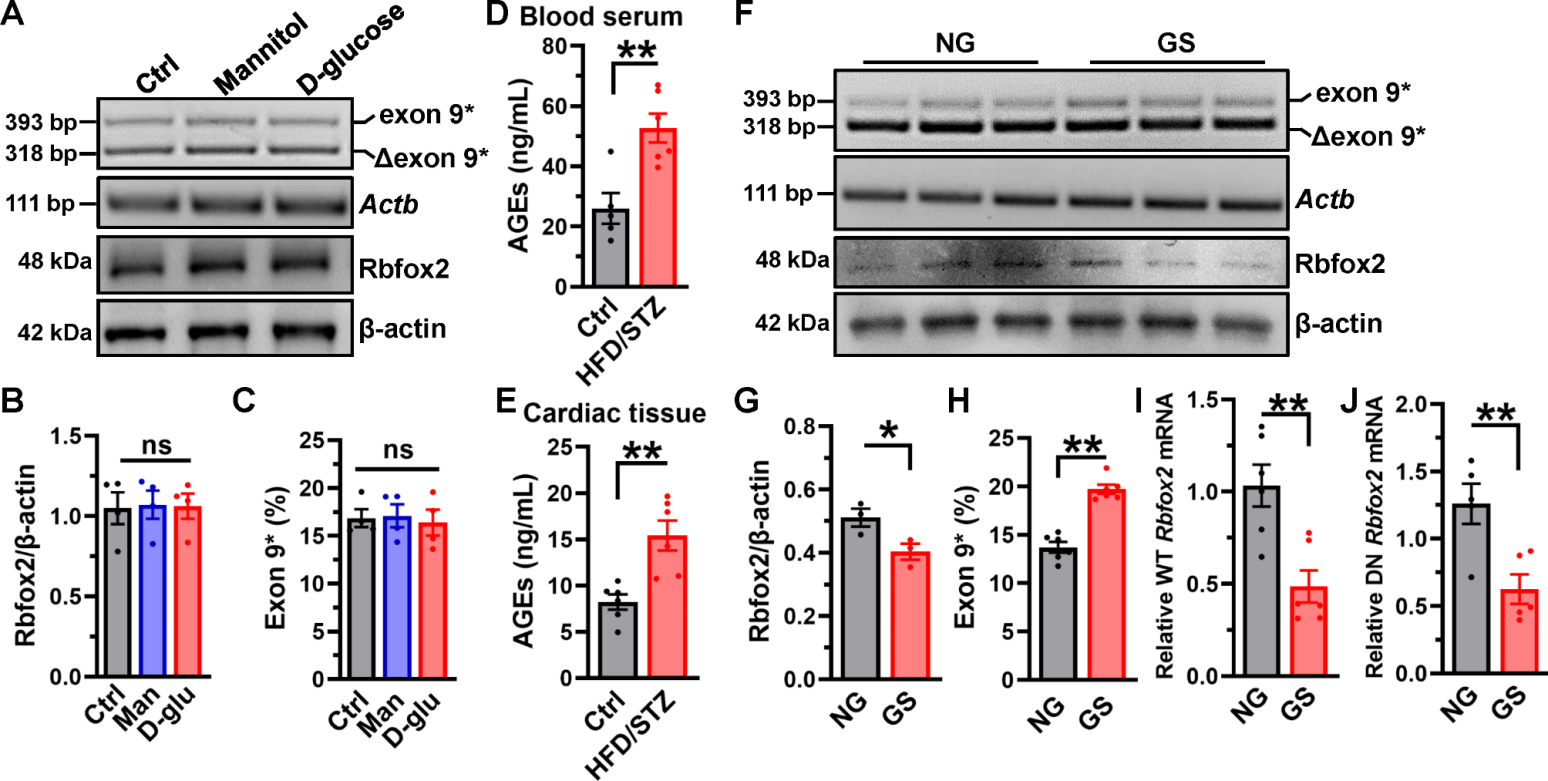
**Glycated serum (GS), not D-glucose, decreases Rbfox2 expression but increases Ca**_**V**_**1.2**_E9* _**channels**. (**A**) NRVMs were treated with mannitol or D-glucose, nontreated cells were set as control. After 48 h incubation, the endogenous expression of Rbfox2 protein was detected by Western blotting, the β-actin was detected as internal control. (**B**) Relative Rbfox2 expression was normalized with β-actin in differentially-treated cells. n = 4 independent experiments. *P* = 0.9854, one-way ANOVA followed by a Tukey’s post hoc test. (**C**) PCR products amplified from cDNA libraries of differentially-treated NRVM were separated on 2.5% agarose gel, and the values of proportion of Ca_V_1.2_E9*_ were analyzed. n = 4 independent experiments. *P* = 0.9109, one-way ANOVA followed by a Tukey’s post hoc test. (**D**) Serum levels of advanced glycation end-products (AGEs) were measured by an ELISA kit in control (n = 5) and HFD/STZ-treated rats (n = 6). **P = 0.0042, unpaired *t* test. (**E**) The levels of AGEs in the lysate of cardiac tissues from different rats’ models (n = 6 each group) were also measured by ELISA. **P = 0.0027, unpaired *t* test. (**F**) NRVMs were treated with 10% non-glycated (NG) or glycated serum (GS) for 48 h, after that the cells were harvested for detecting Ca_V_1.2 alternative exon 9* and expression of Rbfox2 by RT-PCR and Western blotting, respectively. (**G**) Relative expression of Rbfox2 was also analyzed. n = 3 independent experiments. *P = 0.0478, unpaired *t* test. (**H**) The values for percent Ca_V_1.2_E9*_ channels were presented. n = 6 independent experiments. *P* < 0.0001, unpaired *t* test. (**I**-**J**) WT and DN *Rbfox2* mRNA expression were also determined by real-time RT-PCR in NRVMs treated with NG or GS. *Actb* mRNA was measured as internal control. n = 5–6 independent experiments. **P < 0.01, unpaired *t* test. ns indicates no significant differences

Though aberrant expressions of Rbfox2 and Ca_V_1.2 AS were presented in diabetic heart, the underlying regulation mechanisms remained unknown. High blood glucose is a key feature in diabetes, thus we treated the NRVMs with 33.3 mmol/L D-glucose or isosmotic mannitol for 48 h (Fig. 5A). Unexpectedly, the result showed that neither the expression of Rbfox2 nor the proportion of Ca_V_1.2_E9*_ channel was changed after treatment with D-glucose (Fig. 5B-C). Therefore, we speculated that high glucose (HG) may not affect the Rbfox2 expression and Ca_V_1.2 AS.

AGEs are glycated proteins or lipids with long term-exposure to hyperglycemia, which are closely associated with cardiac complications [5]. In the blood serum (Fig. 5D) and lysate of cardiac tissue (Fig. 5E) from HFD/STZ-treated rats, the level of AGEs was significantly increased in comparison to those from controls. Therefore, we used GS, the mimic of AGEs, to incubate with NRVMs for 48 h, the results indicated significantly decreased Rbfox2 expression in comparison to NG-treated cells (Fig. 5F-G). The proportion of Ca_V_1.2_E9*_ channels was increased by ~ 6% after GS treatment (Fig. 5H), however, the proportion of Ca_V_1.2_E33_ channels remained unchanged (Figure S4C-D). Further, we found that both of WT and DN *Rbfox2* mRNA levels are decreased (Fig. 5I-J), which explains the obvious decreased Rbfox2 protein after treatment with GS in cardiomyocytes. Additionally, we found that AGEs application could directly decrease the expression of Rbfox2, and increase the proportion of Ca_V_1.2_E9*_ channels in NRVMs (Figure S5A-C). Moreover, treatment with MGO, a highly reactive dicarbonyl compound forming AGEs [35], also decreased the expression of Rbfox2, but increased the proportion of Ca_V_1.2_E9*_ channels (Figure S5D-F).

### GS treatment shifts window currents of Ca_V_1.2 channel to hyperpolarization in NRVMs

**Fig. 6:**
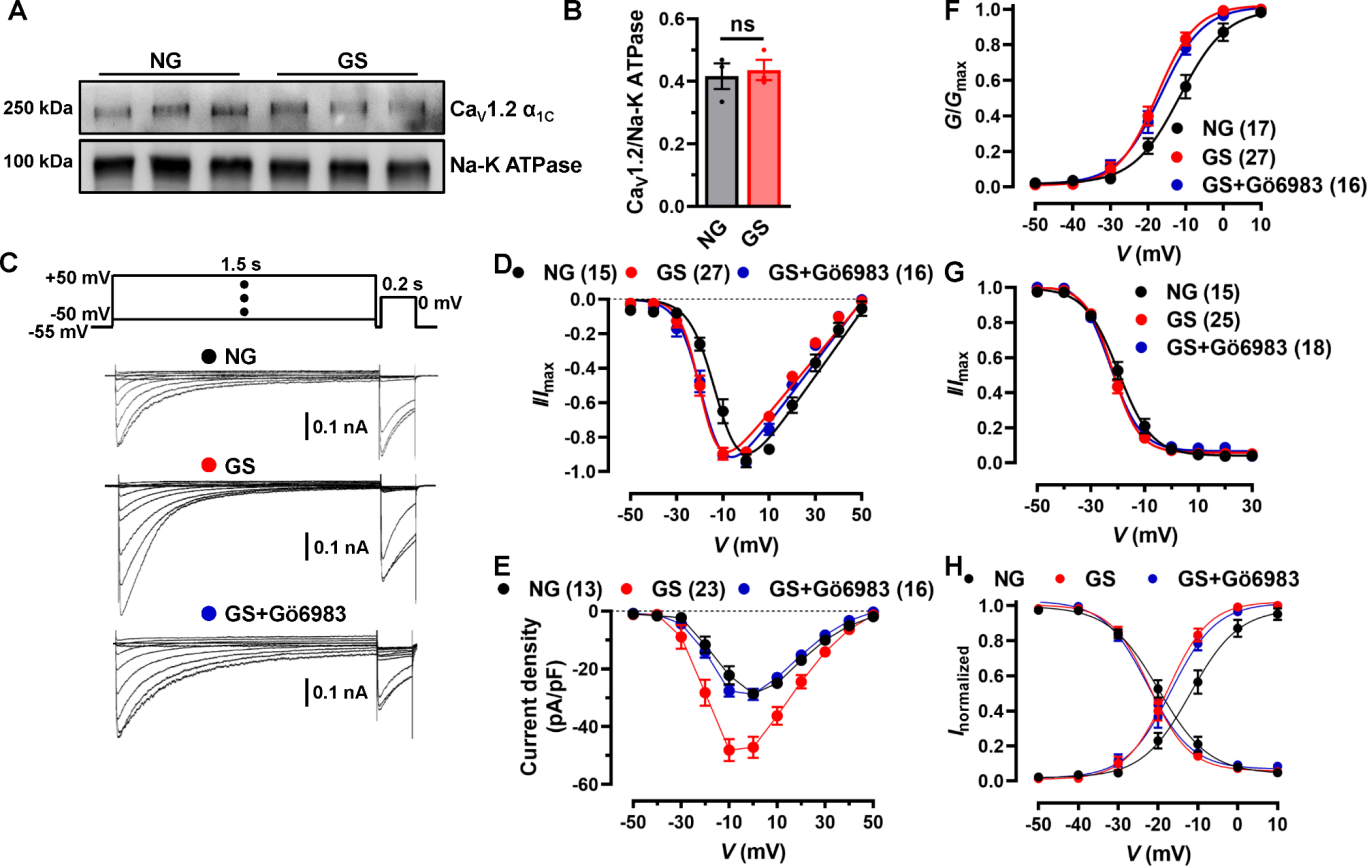
**GS application induces hyperpolarized window currents of Ca**_**V**_**1.2 channel in NRVMs**. (**A**) NRVMs were treated with 10% NG or GS for 48 h, then the membrane protein was extracted. Ca_V_1.2 α_1C_ subunit was detected, and Na-K ATPase was detected as internal control. (**B**) Relative expression of Ca_V_1.2 α_1C_ subunit was normalized with Na-K ATPase. n = 3 independent experiments. *P* = 0.7251, unpaired *t* test. ns indicates no significant differences. (**C**) Ca_V_1.2 channel currents were recorded from NRVMs with NG, GS or GS + Gö6983 treatment in 10 mmol/L Ba^2+^ bath solution. (**D**) *I-V* relationship curves of Ca_V_1.2 channel were recorded under different testing potentials in NRVMs. (**E**) Current densities of Ca_V_1.2 channel in NRVMs treated with NG, GS or GS + Gö6983 were presented. (**F**) Plots of steady-state activation (SSA) curve of Ca_V_1.2 channel were derived from *I*-*V* currents in differentially-treated NRVMs. (**G**) Plots of the steady-state inactivation (SSI) curve were recorded and analyzed in NRVMs. (**H**) Window currents were superimposed from SSI (*f*_*∞*_) and SSA (*d*_*∞*_) curves of NG, GS or GS + Gö6983-treated NRVMs

To further investigate the effects of GS on Ca_V_1.2 channel, we detected the membrane expression of Ca_V_1.2 in NRVMs. Our result indicated that treatment with GS did not affect the membrane expression of Ca_V_1.2 α_1C_ (Fig. 6A-B), which was different from the result in the HFD/STZ-treated rats’ hearts. Next, the electrophysiological functions were analyzed by whole-cell patch clamp. Stepwise voltage stimulations from − 50 to 50 mV were applied to record the currents of Ca_V_1.2 channels in NRVMs (Fig. 6C). The observed *I-V* curve shifted towards left (Fig. 6D, Table S5). The peak current density of NRVMs treated with 10% GS was significantly increased by ~ 2-folds when compared to NG-treated NRVMs (Fig. 6E). We speculated that it might be mediated by protein kinase C (PKC) [32, 36], which is also indicated by KEGG database of AGE-RAGE signaling pathways. Thus, we preincubated the NRVMs with PKC inhibitor Gö6983, and found that Gö6983 could abolish the GS-induced Ca_V_1.2 channel currents in NRVMs (Fig. 6E). As shown in Fig. 6F, the SSA curve was shifted to the hyperpolarized potential, which suggested that Ca_V_1.2 channels on NRVMs treated with GS could open at more negative potentials. Additionally, the SSI curve was also hyperpolarized (Fig. 6G). As expected, the preincubation with Gö6983 didn’t affect GS-induced Ca_V_1.2 channel kinetic changes in NRVMs (Fig. 6F-G). Therefore, the window current of NRVMs treated with GS, superimposed from SSA and SSI curves, was shifted toward hyperpolarization compared to those with NG-treated NRVMs (Fig. 6H, Table S5). Collectively, our data indicated that GS treatment hyperpolarizes the channel kinetics by modulating the proportion of Ca_V_1.2_E9*_ channels in NRVMs, and we hypothesized that cardiomyocyte hypertrophy under GS treatment is related to the change of kinetics of cardiac Ca_V_1.2 channels.

### GS treatment increases [Ca^2+^]_i_ and induces cardiomyocyte hypertrophy in NRVMs

**Fig. 7:**
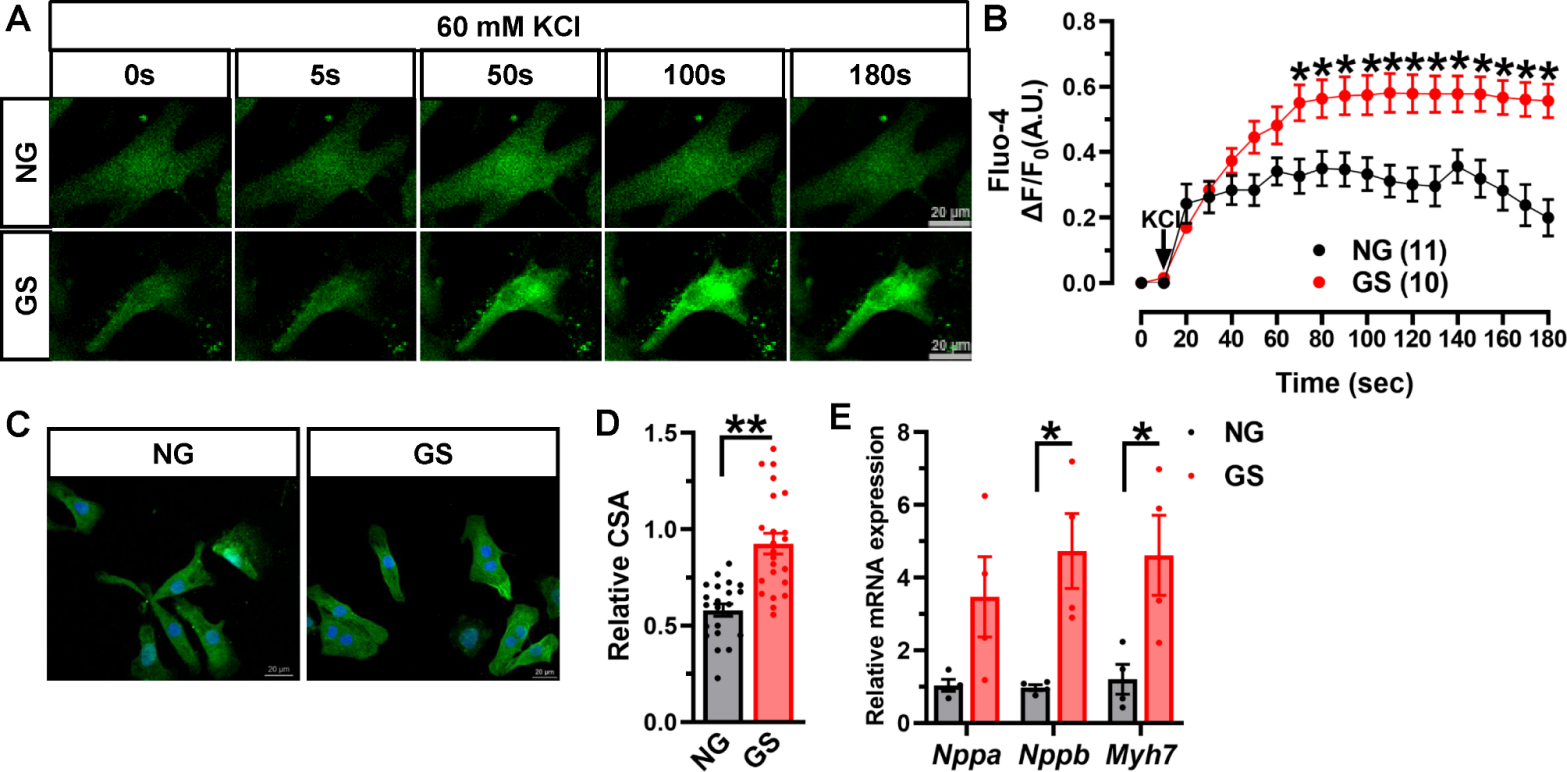
**GS application induces cardiomyocyte hypertrophy**. (**A**) NRVMs treated with 10% NG or GS, real-time [Ca^2+^]_i_ was measured by Ca^2+^ fluorescence indicator Fluo-4 AM, and the fluorescent intensity was monitored by time series scanning mode under a confocal microscope. Δ[Ca^2+^]_i_ fluorescence intensities were measured by dividing the changes in the fluorescent signal by the average resting fluorescence. (**B**) Plots of time course of fluorescent intensity after application with NG (n = 11 cells) or GS (n = 10 cells) in NRVMs from 3 independent experiments, Δ[Ca^2+^]_i_ was presented as Δ*F*/*F*_0_. *P < 0.05, two-way ANOVA followed by Sidak’s multiple comparisons test. (**C**) Representative images of NRVMs treated with NG or GS showed immunofluorescence staining by using anti-α-actinin antibody to determine cell surface area (CSA). (**D**) Analyzed CSA was shown as scatter plots in NG (n = 22 cells) or GS-treated NRVMs (n = 23 cells) from 3 independent experiments. **P < 0.0001, unpaired *t* test with Welch’s correction. (**E**) Rat *Nppa, Nppb* and *Myh7* mRNAs were determined by real-time RT-PCR in differentially-treated NRVMs, rat *Actb* mRNA was detected as internal control. n = 4 independent experiments. *P < 0.05, unpaired *t* test with or without Welch’s correction

Since GS treatment hyperpolarized Ca_V_1.2 window current in cardiomyocytes, which in turn facilitated the channel function. Next, we monitored [Ca^2+^]_i_ in NRVMs by using Fluo-4 AM indicator (Fig. 7A). Extracellular K^+^ could directly depolarize cell membranes and activate Ca_V_1.2 channels on cardiomyocytes, initiating the CIRC. In comparison to NG-treated NRVMs, we found that treatment with GS further raises K^+^-induced [Ca^2+^]_i_ elevation in NRVMs (Fig. 7B). Though we cannot exclude the other target players, like ryanodine receptor 2 (RyR2) and sarcoplasmic/endoplasmic reticulum Ca^2+^ ATPase 2a (SERCA2a) [37], mediate GS-induced [Ca^2+^]_i_ elevation, our data showed that GS-induced hyperpolarized Ca_V_1.2 channel kinetics at least partly raises K^+^-induced [Ca^2+^]_i_ elevation in NRVMs.

To determine whether AGEs induce cardiomyocyte hypertrophy, NRVMs were treated with 10% GS for 48 h, which showed marked increments of the CSA of cardiomyocytes (Fig. 7C-D). Consistent with animal results, the expression level of *Nppa* mRNA was slightly increased, and the levels of *Nppb* and *Myh7* mRNA were obviously increased in GS-treated cells compared to NG-treated ones (Fig. 7E). Previous results including ours indicated that increased [Ca^2+^]_i_ induced by overactivated Ca_V_1.2 channel perturbs the homeostasis of intracellular calcium, which triggers cardiac hypertrophy [19, 38]. These results suggested that GS might induce cardiomyocyte hypertrophy by modulating Ca_V_1.2 channel-mediated [Ca^2+^]_i_ elevation.

### Knockdown of Rbfox2 directly hyperpolarize the window currents of Ca_V_1.2 channel in NRVMs

**Fig. 8:**
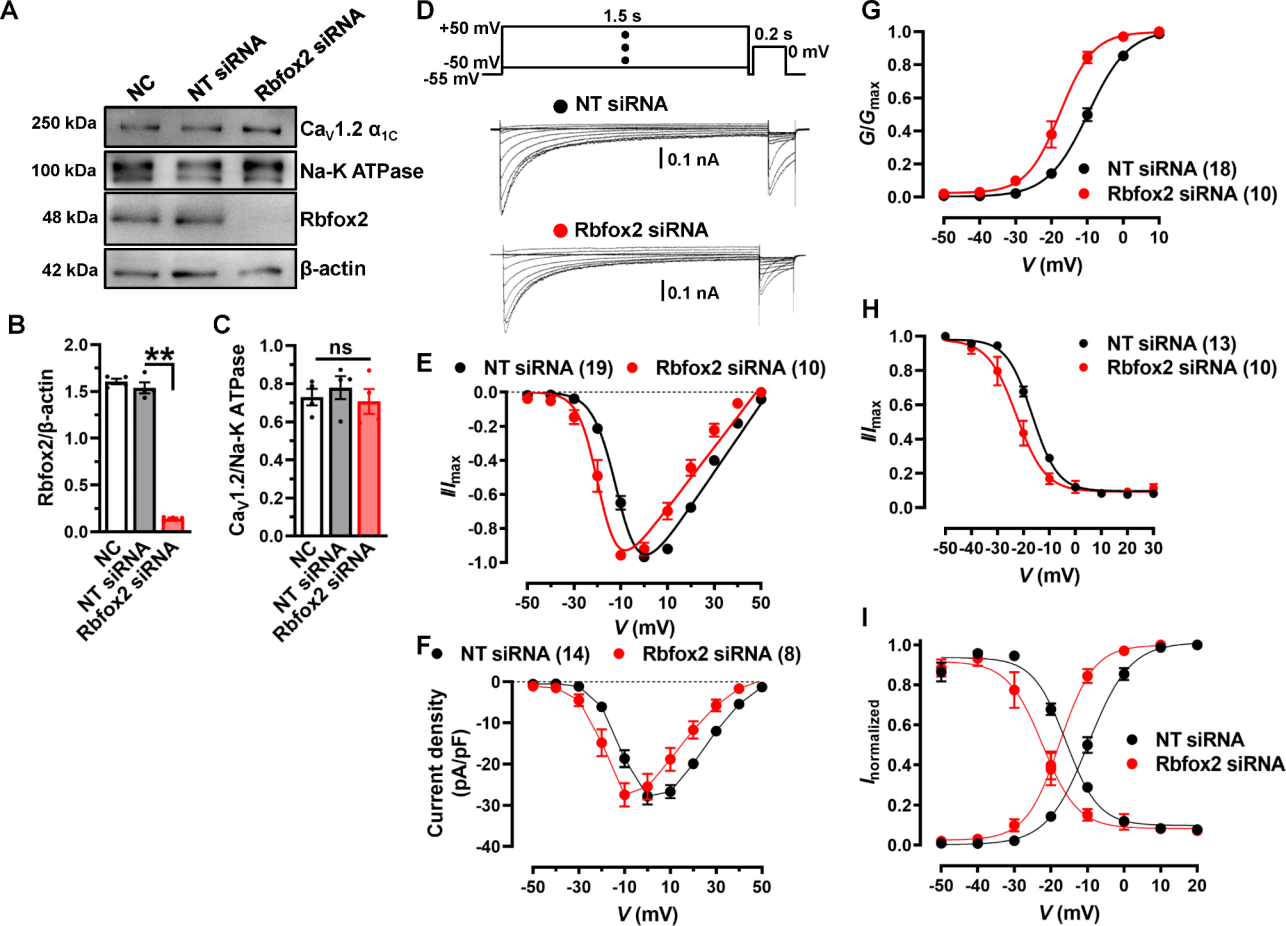
**Knockdown of Rbfox2 hyperpolarizes window currents of Ca**_**V**_**1.2 channel in NRVMs**. (**A**) The protein expression of Rbfox2 and β-actin were detected in whole-cell lysate of isolated NRVMs by using Western blotting after transfecting with NT or Rbfox2 siRNAs. The membrane protein was also extracted, and membrane expression of Ca_V_1.2 α_1C_ was checked, Na-K ATPase was detected as a membrane loading control. (**B**) Relative expression level of Rbfox2 was normalized with β-actin in differentially transfected cells, and presented as a bar chart. n = 4 independent experiments. **P < 0.001, one-way ANOVA followed by a Tukey’s post hoc test. (**C**) Relative Ca_V_1.2 α_1C_ membrane expression was normalized with Na-K ATPase in differentially-transfected cells. n = 4 independent experiments. *P* = 0.6679, one-way ANOVA followed by a Tukey’s post hoc test. (**D**) Raw traces of Ca_V_1.2 whole-cell calcium current recorded from NRVMs treated with NT or Rbfox2 siRNA in 10 mmol/L Ba^2+^ external solution. (**E**) *I-V* relationship of calcium channel current recorded under the different testing potential, increased from − 50 to 50 mV in NRVMs transfected with NT or Rbfox2 siRNA. (**F**) Ca_V_1.2 channel current density in NRVMs was analyzed after transfected with NT or Rbfox2 siRNA. (**H**) Plots of steady-state activation (SSA) curve of Ca_V_1.2 channel were analyzed from *I-V* currents in NT or Rbfox2 siRNA-treated NRVMs. (**H**) Plots of the steady-state inactivation (SSI) was also recorded in NRVMs. (**I**) Ca_V_1.2 window currents were superimposed from SSI (*f*_*∞*_) and SSA (*d*_*∞*_) curves of NRVMs

We found that application with GS causes a decrease in Rbfox2 protein expression and an increase in the proportion of Ca_V_1.2_E9*_ channels, and left-ward shifts Ca_V_1.2 in NRVMs. To further study the relevance of Rbfox2-mediated AS and Ca_V_1.2 channel functions in NRVMs, we used siRNA approach to knock down Rbfox2 expression, but didn’t affect the membrane expression of Ca_V_1.2 channels (Fig. 8A-C). Whole-cell patch clamp was utilized to measure the channel currents after knocking down Rbfox2 (Fig. 8D). Here, we observed that the *I-V* curve of NRVMs treated with Rbfox2 siRNA shifted toward left as against to non-targeting siRNA-treated NRVMs (Fig. 8E, Table S6). But the peak current density of Ca_V_1.2 channels remained unchanged (Fig. 8F). Furthermore, our results showed that both of the SSA and SSI curves shift toward left (Fig. 8G-H, Table S6), making the window currents hyperpolarized-shift after knocking down Rbfox2 (Fig. 8I). In summary, our data revealed that Rbfox2 indeed directly affects the electrophysiological properties of Ca_V_1.2 channel through regulating AS events in NRVMs.

### Knockdown of Rbfox2 induces elevated [Ca^2+^]_i_ and hypertrophy of NRVMs

**Fig. 9:**
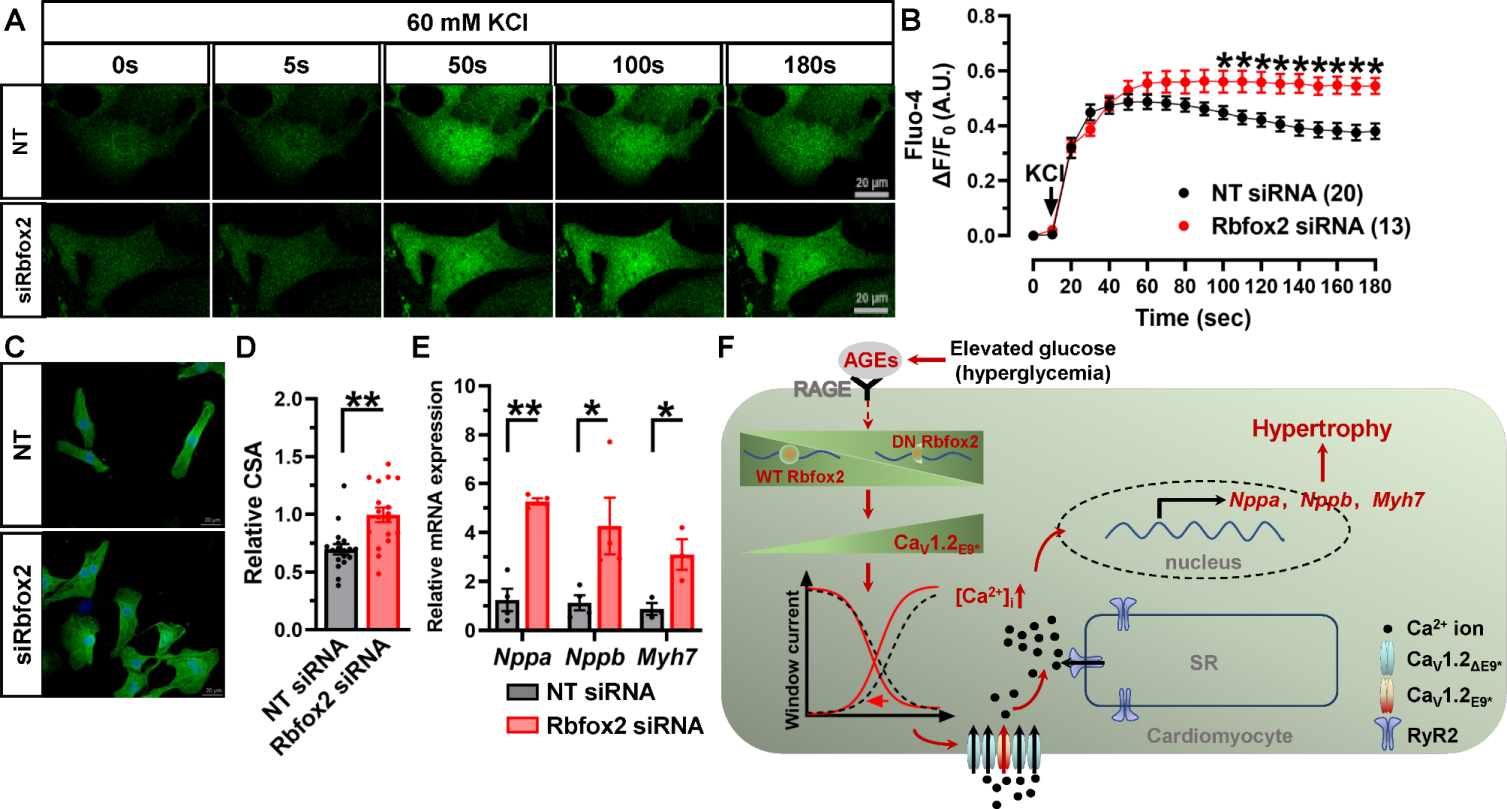
**Knockdown of Rbfox2 induces cardiomyocyte hypertrophy**. (**A**) NRVMs treated with NT or Rbfox2 siRNAs, real-time [Ca^2+^]_i_ was measured by Ca^2+^ fluorescence indicator Fluo-4 AM, Δ[Ca^2+^]_i_ fluorescence intensities were measured by dividing the changes in the fluorescent signal by the average resting fluorescence. (**B**) Plots of time course of fluorescent intensity after application with NT (n = 20 cells) or Rbfox2 siRNAs (n = 13 cells) in NRVMs from 3 independent experiments, Δ[Ca^2+^]_i_ was presented as Δ*F*/*F*_0_. *P < 0.05, two-way ANOVA followed by Sidak’s multiple comparisons test. (**C**) Representative images of NRVMs transfected with NT or Rbfox2 siRNA, immunofluorescence staining by using anti-a-actinin antibody was applied to determine cell surface area (CSA). (**D**) Analyzed CSA was shown as a bar chart in NT (n = 18 cells) or Rbfox2 siRNA (n = 18 cells) transfected NRVMs from 3 independent experiments. **P = 0.0005, unpaired *t* test. (**E**) Rat *Nppa, Nppb* and *Myh7* mRNAs were determined by real-time RT-PCR in differentially-treated NRVMs, rat *Actb* mRNA was detected as internal control. n = 3–4 independent experiments. *P < 0.05, **P < 0.01, unpaired *t* test. (**F**) Illustration of main findings in this study. Under diabetic hyperglycemia, AGEs, not glucose, induces aberrant expression of Rbfox2, which upregulates Ca_V_1.2_E9*_ channels in cardiomyocyte. AGEs/Rbfox2-mediated AS hyperpolarizes cardiac Ca_V_1.2 window currents, elevates K^+^-triggered [Ca^2+^]_i_ and promotes fetal genes’ transcription, finally induces cardiomyocyte hypertrophy. RAGE, receptor for advanced glycation end-products; RyR2, ryanodine receptor 2; SR, sarcoplasmic reticulum

It has been previously shown that ablation of Rbfox2 in adult mouse hearts impairs excitation coupling, and targeting Rbfox2 affected calcium homeostasis in diabetic hearts [29, 39]. This made us to explore whether Rbfox2 regulates cardiomyocyte intracellular calcium homeostasis by affecting Ca_V_1.2-mediated calcium influx. Here, we found knockdown of Rbfox2 raises the K^+^-triggered [Ca^2+^]_i_ elevation in NRVMs (Fig. 9A-B), strongly implying that Rbfox2 can affect the homeostasis of Ca^2+^ in cardiomyocytes.

We next investigated whether manipulation of Rbfox2 could directly induce cardiomyocyte hypertrophy. NRVMs were transfected with siRNA to achieve specifically knockdown the endogenous expression of Rbfox2. After 48 h incubation, the CSA was enlarged (Fig. 9C-D), and the mRNA levels of hypertrophic genes, including *Nppa*, *Nppb* and *Myh7*, were dramatically increased (Fig. 9E). These confirmed diminished Rbfox2 could induce cardiomyocyte hypertrophy, which we thought might be mediated by modulating cardiac Ca_V_1.2 AS.

## Discussion

Ca^2+^ influx from cardiac Ca_V_1.2 channel plays a significant role in the physiological processes of cardiomyocytes [6]. Imbalanced Ca^2+^ homeostasis induced by dysfunctional Ca_V_1.2 channel is closely associated with cardiomyopathies. Here, we found that (1) Ca_V_1.2 channels with alternative exon 9*, but not those with exon 8a or exon 33, are specifically increased in the heart from diabetic rats; (2) AGEs, not glucose, induces aberrant expression of Rbfox2, and upregulates Ca_V_1.2_E9*_ channels in cardiomyocyte; (3) AGEs/Rbfox2-mediated exon 9* insertion hyperpolarizes cardiac Ca_V_1.2 window currents; and (4) AGEs application or knockdown of Rbfox2 increases [Ca^2+^]_i_, enlarges CSA and triggers the hypertrophic genes transcription in cardiomyocytes, which mediates cardiomyocyte hypertrophy (Fig. 9F).

As a hub for Ca^2+^ handling, aberrant splicing of Ca_V_1.2 channel is presented in different heart diseases, like myocardial infraction [18], cardiac hypertrophy [19] and ischemic or dilated cardiomyopathy [14]. Here, Ca_V_1.2 channel is found to be aberrantly spliced in diabetic heart with a specific manner, that only Ca_V_1.2 alternative exon 9* was increased, but exon 8/8a or exon 33 remained unchanged. HG is known to work as a pathological factor in diabetes, which promotes the development of cardiovascular implications. In arterial smooth muscle, HG facilitated the Ca_V_1.2 functions by protein phosphorylation, which increased the transiently vascular constriction under diabetic hyperglycemia. Thus, we initially thought HG may mediate the aberrant splicing of Ca_V_1.2 channel in the diabetic heart. Unexpectedly, the treatment with HG didn’t affect Ca_V_1.2 alternative exon 9* in cardiomyocytes, indicating that HG might not make the direct effects on Ca_V_1.2 AS events. Long-term HG interacts with proteins, lipids, and/or nucleic acids in blood to generate lots of AGEs [40], which may be the initiator of evil in the aberrant splicing of Ca_V_1.2 in diabetic hearts. We found treatment with AGEs could increase the proportion of Ca_V_1.2_E9*_ channels in cardiomyocytes, same with the pattern found in the diabetic heart. Thus, we concluded that AGEs make direct effects on Ca_V_1.2 AS, properly attributing to its metabolic memory [41, 42].

Rbfox2 is known to contribute to heart development [26], and under pathological conditions, diminished Rbfox2 contributes to pressure-overload-induced heart failure [39] and cardiac decompensation leading to heart failure [27]. Here, we identified that the expression of Rbfox2 protein is increased presumably owing to upregulation of DN Rbfox2, as WT *Rbfox2* mRNA was decreased in the hearts from diabetic rats, which are consistent with previous findings [29]. These are possibly caused by the stimulatory signaling inducing the increased DN Rbfox2 in the whole heart, but its detailed mechanisms remained yet to be further investigated. *In-vitro* experiment found GS application decreases both WT and DN *Rbfox2* mRNAs in NRVMs. There are 3 UGCAUG elements surrounding exon 9* of *Cacna1c* gene, and Rbfox protein prefers to bind with the upstream of cassette exon 9*, which depresses the expression of exon 9* of Ca_V_1.2 channel during neuronal development [24]. Our results indicated that Rbfox2 knockdown increases Ca_V_1.2_E9*_ channels in cardiomyocytes, in line with our previous findings in vascular smooth muscle [16]. Moreover, overexpression of DN Rbfox2 increased Ca_V_1.2_E9*_ channels, owing to the inhibition of WT Rbfox2 splicing activity [34]. Therefore, increased proportion of Ca_V_1.2_E9*_ channels in the heart from diabetic rats were attributed to dual regulatory methods, downregulated WT Rbfox2 and upregulated DN Rbfox2. Though we cannot exclude the possibility that Rbfox2 regulates other genes AS events in heart, like guanine exchange factors of Rho GTPase family proteins [26], the presented data revealed that Rbfox2 indeed induce cardiac hypertrophy, which is, at least partly, mediated by regulating Ca_V_1.2 AS events.

Ca_V_1.2 alternative exon 9* is 25 amino acids in size and behind exon 9, where has α_1_-subunit interaction domain in α_1C_ I-II loop [15]. Ca_V_1.2 channel with exon 9* was found to hyperpolarize the window currents in human embryonic kidney-293 cells [20], probably because of the structural change in the endoplasmic reticulum export signaling site of Ca_V_1.2 I-II loop, where it binds to β subunit [15, 43]. Furthermore, insertion of exon 9* increased basal Ca_V_1.2 channel open probability and conductance by stabilizing the rigid α_1_-subunit interaction domain helical linker, or affecting the function of the voltage sensor in domain II [44]. Increased Ca_V_1.2_E9*_ channels are found in diabetic heart, which is attributed by AGEs-induced downregulation of Rbfox2, and hyperpolarized window current of Ca_V_1.2_E9*_ channels make it easier to be opened at the potentials closer to resting membrane potential. As we previously found, function of Ca_V_1.2 channel was enhanced in isoproterenol-induced cardiomyocyte hypertrophy, and targeting Ca_V_1.2 channel could inhibit the hypertrophic signaling in the heart [19]. Therefore, the facilitated cardiac Ca_V_1.2_E9*_ channel should play a significant role in the process of development in diabetes-induced cardiac hypertrophy.

Ca^2+^ influx through Ca_V_1.2 channel triggers Ca^2+^ ions to be released from sarcoplasmic reticulum to cytosol by CICR, and transiently increasing the [Ca^2+^]_i_, which leads to cardiac contraction [45, 46]. Besides that, increased [Ca^2+^]_i_ will initialize genes transcription in cardiomyocyte by excitation-transcription coupling [7]. Hyperpolarized window currents of Ca_V_1.2 channels is deduced to increase Ca^2+^ influx, which elevates [Ca^2+^]_i_ [20]. This result was proved by the finding that GS application increases [Ca^2+^]_i_ in cardiomyocytes, which we thought is at least partly attributed to Rbfox2-mediated Ca_V_1.2 AS. In the heart, increased Ca^2+^ influx through Ca_V_1.2 channel raised fetal genes transcription, leading to cardiomyocyte hypertrophy [19, 38]. Here, GS treatment or Rbfox2 knockdown could increase CSA and hypertrophic genes transcription. As GS directly decreased Rbfox2 expression in cardiomyocytes, reasonably, GS might induce cardiomyocyte hypertrophy by reducing the expression of Rbfox2.

Though the inhibition of enhanced Ca_V_1.2 channel activity by LTCC blockers might have benefits in cardiac remodeling against cardiomyopathies in the murine model [47, 48], the effects of these drugs remain controversial in the patients with cardiomyopathy [49]. We think that there are several possibilities for these findings. First, LTCC blockers, used clinically for decades, have none or very little selectivity for the different subtypes of LTCCs [50, 51], such as Ca_V_1.3 calcium channels which are also expressed in the heart. Second, Ca_V_1.2 channels are widely expressed in different organs or systems [52], therefore the usage of LTCC blockers could produce adverse effects by targeting Ca_V_1.2 channels on the other tissues including blood vessels and the brain. Therefore, targeting Ca_V_1.2 splice isoform which specifically expressed in diseased heart might be a promising approach to manage cardiomyopathies [11]. Here, increased Ca_V_1.2_E9*_ channels produced enhanced channel functioning in diabetic hearts, we think by targeting Ca_V_1.2 exon 9* or its regulatory factor Rbfox2 is possibly an optimal idea to manage DCM, which requires further investigations.

## Conclusions

From the new dimension, we identified for the first time that Ca_V_1.2 channel is aberrantly spliced in diabetic heart with a specific manner, which enhances the channel function, and this effect was mediated by AGEs-induced Rbfox2 downregulation under diabetic hyperglycemia. Therefore, targeting Rbfox2 to reset the aberrantly spliced Ca_V_1.2 channel might be a promising therapeutic approach in diabetes-induced cardiac hypertrophy.

## Electronic supplementary material

Below is the link to the electronic supplementary material.

Supplementary Material 1

## Data Availability

The datasets used and/or analysed during the current study are available from the corresponding author on reasonable request.

## Abbreviations

AGEs: Advanced glycation end-products
AS: Alternative splicing
[Ca^2+^]_i_: Intracellular Ca^2+^ concentration
CICR: Calcium-induced calcium release
CSA: Cell surface area
DCM: Diabetic cardiomyopathy
DN: Dominate-negative
FBS: Fetal bovine serum
FS: Fractional shortening
GS: Glycated serum
HFD: High-fat diet
HG: High glucose
*I–V*: Current-voltage
LTCC: L-type calcium channel
LVEF: Left ventricle ejection fraction
MGO: Methylglyoxal
NG: Non-glycated serum
NRVM: Neonatal rat ventricular myocyte
RAGE: Receptor for advanced glycation end-products
RyR2: Ryanodine receptor 2
SD: Sprague-Dawley
S.E.M.: Standard error of mean
SERCA2a: Sarcoplasmic/endoplasmic reticulum Ca^2+^ ATPase 2a
SSA: Steady-state activation
SSI: Steady-state inactivation
STZ: Streptozotocin
WT: Wild-type
