# Supplementary material for: Dysregulated Rbfox2 produces aberrant splicing of CaV1.2 calcium channel in diabetes-induced cardiac hypertrophy
Source: Cardiovasc Diabetol. 2023 Jul 6;22:168. doi: 10.1186/s12933-023-01894-5 (PMC10324275; doi:10.1186/s12933-023-01894-5)
Supplement: Supplementary file 1 — Supplementary Material 1 [file 12933_2023_1894_MOESM1_ESM.pdf]

Supplementary Information

Dysregulated Rbfox2 produces aberrant splicing of $Ca_V1.2$ calcium channel in diabetes-induced cardiac hypertrophy

Pengpeng Li, Dongxia Qin, Tiange Chen, Wei Hou, Xinyu Song, Shumin Yin, Miaomiao Song, W.C. Hewith A. Fernando, Xiaojie Chen, Yu Sun*, Juejin Wang*

Key Laboratory of Targeted Intervention of Cardiovascular Disease, Department of Physiology, Nanjing Medical University, Nanjing, Jiangsu, China

Short title: Rbfox2 and $Ca_V1.2$ AS in diabetic heart

*Correspondence:

Juejin Wang, Department of Physiology, Nanjing Medical University, 101 Longmian Ave, Nanjing 211166, Jiangsu, China. Email: juejinwang@njmu.edu.cn

or Yu Sun, Department of Physiology, Nanjing Medical University, 101 Longmian Ave, Nanjing 211166, Jiangsu, China. Email: sunyu716@njmu.edu.cn

Table S1. Primer sequences for PCR

Target genes	Oligonucleotide primers (5'-3')	Product length (bp)
Rat WT *Rbfox2**	FP: AGGGCCGTAAAATCGAGGTG<br>RP: GCCGTACACAGCTCCAACTA	116
Rat DN *Rbfox2**	FP: CGGTTCCAAGGTGAATAATGC<br>RP: TATAACTCAGGGCCGTACACA	120
Rat *Cacna1c* exon 9*/Δ9*	FP: CCGTGTGCAAGCCCGGGTGGG<br>RP: GGATTTGGAGATCCGGTGG	587 (9*)<br>512 (Δ9*)
Rat *Cacna1c* exon 33/Δ33	FP: GCCTCTTCACGGTGGAG<br>RP: TCCCAATCACTGCATAGATAA	357 (33)<br>324 (Δ33)
Rat *Nppa**	FP: GGCTTCTTCCTCTTCCTGG<br>RP: CCGCTTCATCGGTCTGC	184
Rat *Nppb**	FP: CTCAAAGGACCAAGGCCCTAC<br>RP: AACCTCAGCCCGTCACAGC	180
Rat *Myh7**	FP: CTCAGTCATGGCGGATCGAGA<br>RP: TCACCGTCTTGCCATTCTCT	215
Rat *Actb**	FP: CGAGTACAACCTTCTTGCAGC<br>RP: AGTCCTTCTGACCCATACCCA	212
Rat *Actb*	FP: ACCCGCGAGTACAACCTTCT<br>RP: CATGCCGGAGCCGTTGTC	111

*These primers were used for real-time PCR.

Table S2. Sequences of siRNAs targeting with rat Rbfox2 mRNA

siRNAs	Sense/antisense	Sequences
#1 siRNA	Sense<br>Antisense	5′ GCCACACACUCAGGACUAUdTdT 3′<br>3′ dTdTCGGUGUGUGAGUCCUGAUA 5′
#2 siRNA	Sense<br>Antisense	5′ GGAUUCGGGUUCGUAACUUdTdT 3′<br>3′ dTdTCCUAAGCCCAAGCAUUGAA 5′
#3 siRNA	Sense<br>Antisense	5′ GGAUAUGCAGCCUACAGAUdTdT 3′<br>3′ dTdTCCUAUACGUCGGAUGUCUA 5′

Table S3. Baseline characteristics of control or HFD/STZ-treated rats

Rats	Age (wk)	HW (g)	BW (g)	HW/BW (mg/g)	TL (cm)	HW/TL (g/cm)	n
Control	16	1.09±0.038	447.6±12.8	2.45±0.10	4.3±0.04	0.25±0.06	6
HFD/STZ-treated rats	16	1.30±0.035**	328.0±8.61**	3.99±0.12**	4.4±0.05	0.29±0.06	6

HW: heart weight; BW: body weight; TL: tibial length. ***P*<0.01 versus control, unpaired *t* test.

Table S4. Echocardiographic characteristics of 16-weeks-old control or HFD/STZ-treated rats

	Control rats (n=6)	HFD/STZ-treated rats (n=6)
IVS;d (mm)	1.57±0.042	1.51±0.06
IVS;s (mm)	2.55±0.16	2.44±0.16
LVID;d (mm)	7.63±0.39	9.19±0.22**
LVID;s (mm)	4.45±0.37	5.68±0.19*
LVPW;d (mm)	1.69±0.07	1.68±0.09
LVPW;s (mm)	2.84±0.10	2.69±0.13
LVEF (%)	70.97±2.96	68.07±2.58
FS (%)	42.05±2.62	39.95±2.06
LV V ol;d (μL)	315.2±36.60	463.60±28.21**
LV V ol;s (μL)	94.48±17.12	148.50±14.84*
E/A	1.64±0.057	1.35±0.07**

IVS, interventricular septum dimension at end diastole (d) or systole (s); LVID, left ventricle internal dimension; LVPW, left ventricle posterior wall thickness; LVEF, left ventricle ejection fraction; FS, fraction shortening; E/A, the ratio between early diastolic mitral flow velocity (E) and late diastolic mitral flow velocity. **P*<0.05, ***P*<0.01 versus control rats, unpaired *t* test.

Table S5. Summary of Cav1.2 channel electrophysiological characteristics of NG, GS or GS plus Gö6983-treated NRVMs

	$V_{0.5}$ (mV)	E_{rev} (mV)	k	n
NG	-11.59±0.90	53.73±1.33	-5.64±0.65	15
GS	-19.20±0.52**	50.97±0.99	-4.30±0.47	27
GS+Gö6983	-18.09±0.66**	50.62±1.03	-5.13±0.51	16
	$V_{0.5,act}$ (mV)	n	$V_{0.5,inact}$ (mV)	n
NG	-11.53±1.11	17	-19.77±0.65	15
GS	-17.61±0.86**	27	-22.00±0.47*	25
GS+Gö6983	-16.67±0.95**	16	-22.71±0.57**	18

$V_{0.5}$: half-activation potential, E_{rev}: reversal potential, k: slope rate. $V_{0.5,act}$: half-activation potential of activation curve, $V_{0.5,inact}$: half-inactivation potential of inactivation curve. *P<0.05, **P<0.01 versus NG-treated NRVMs, one-way ANOVA followed by a Tukey's post hoc test.

Table S6. Summary of Cav1.2 channel electrophysiological characteristics of NT or Rbfox2 siRNAs-treated NRVMs

	$V_{0.5}$ (mV)	E_{rev} (mV)	k	n
NT siRNA	-10.54±0.39	53.41±0.64	-5.05±0.30	19
Rbfox2 siRNA	-18.79±0.78**	48.05±1.45**	-4.30±0.69	10
	$V_{0.5,act}$ (mV)	n	$V_{0.5,inact}$ (mV)	n
NT siRNA	-9.74±0.53	18	-16.59±0.38	13
Rbfox2 siRNA	-17.53±0.90**	10	-22.65±1.27**	10

$V_{0.5}$: half-activation potential, E_{rev}: reversal potential, k: slope rate. $V_{0.5,act}$: half-activation potential of activation curve, $V_{0.5,inact}$: half-inactivation potential of inactivation curve. **P<0.01 versus NT siRNAs-treated NRVMs, Student t test.

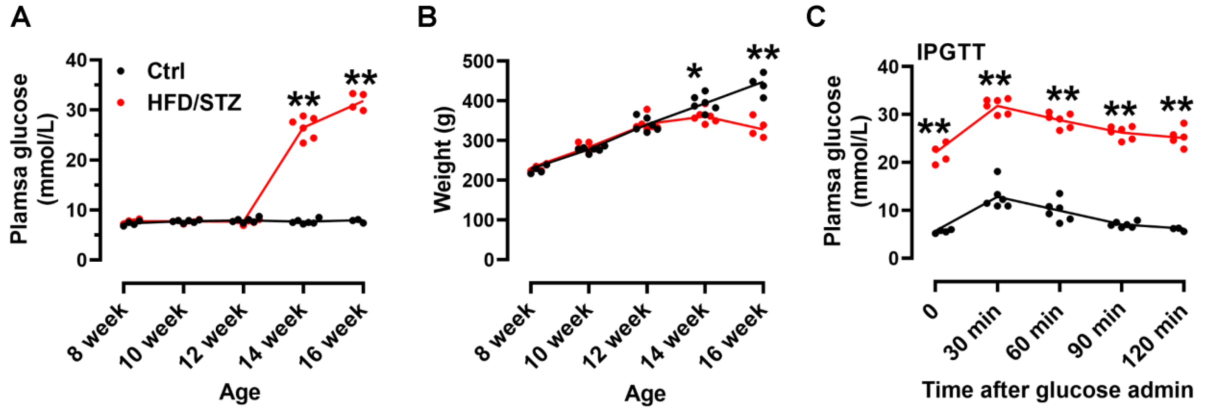

Figure S1. HFD/STZ-treated rats show higher blood glucose and impaired glucose tolerance. (A) Levels of fasting blood glucose were checked from 8-weeks-old to 16-weeks old in control and HFD/STZ-treated rats. (B) Body weights of control and HFD/STZ-treated rats were also detected every two weeks. (C) Intraperitoneal glucose tolerance test (IPGTT) was performed by measuring levels of blood glucose after intraperitoneal injection of D-glucose (2 g/kg). n=6 rats for each group. **P*<0.05, ***P*<0.01, two-way ANOVA followed by Sidak's multiple comparisons test.

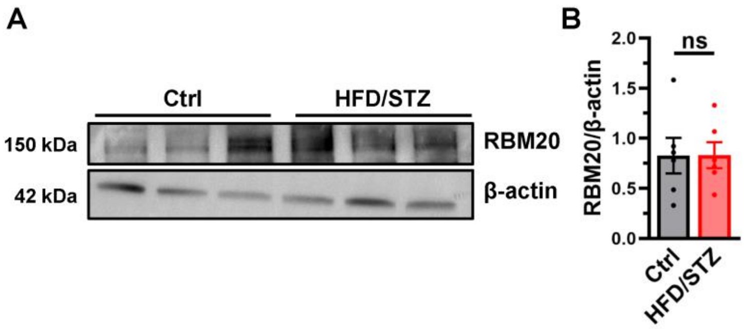

Figure S2. Expressions of RBM20 protein are not changed in the hearts from control or HFD/STZ-treated rats. (A) Expression of RBM20 was detected in whole-cell lysate of heart tissue by using Western blotting, and β-actin was measured as internal control. (B) Relative RBM20 expression was normalized with β-actin. n=6 rats for each group. *P*=0.9803, unpaired *t* test. ns indicates no significant differences.

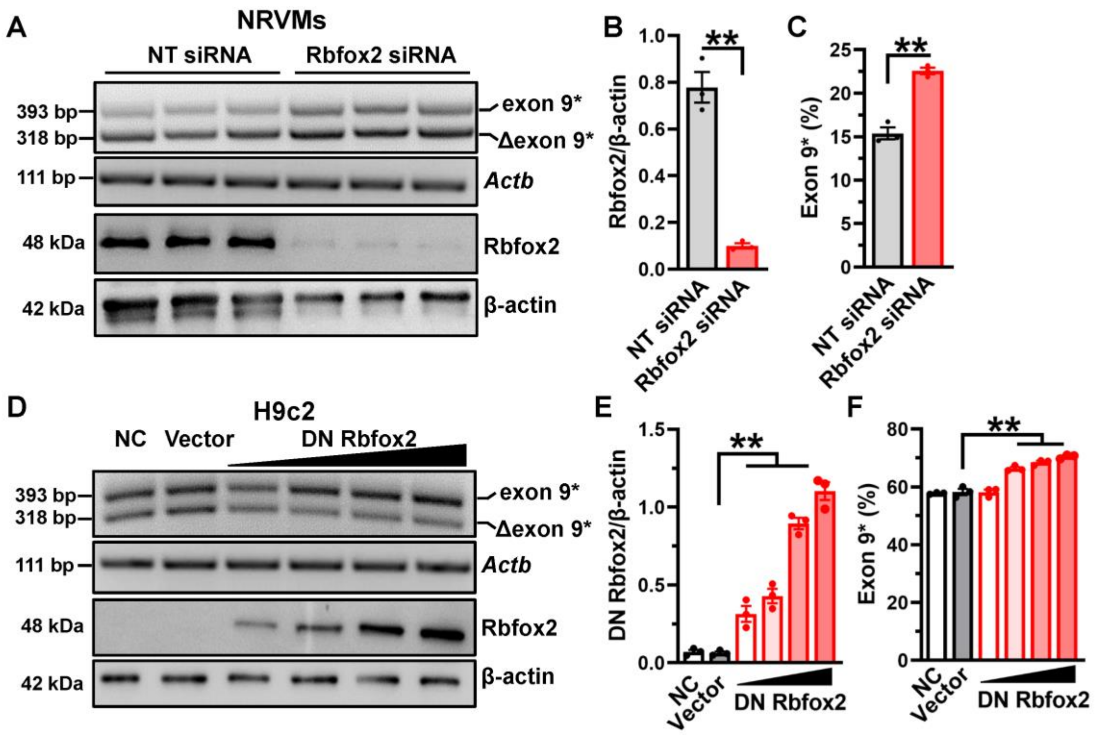

Figure S3. Rbfox2 dynamically modulates Cav1.2 alternative exon 9* in cardiomyocyte. (A) NRVMs were transfected with non-targeting (NT) or Rbfox2 siRNA for 48 hr. The endogenous expression of Rbfox2 protein was detected by Western blotting, β-actin was detected as internal control. PCR products of Cav1.2$_{E9*}$ channels were amplified from cDNA libraries and separated on 2.5% agarose gel. *Actb* mRNA was detected as internal control. (B) Relative expression of Rbfox2 was normalized to β-actin in differentially-treated NRVMs. n=3 independent experiments. **P=0.0005, unpaired t test. (C) The values for percent exon 9* inclusion was the upper band intensity divided by the summed intensities of upper and lower bands. n=3 independent experiments. **P=0.0008, unpaired t test. (D) H9c2 cells were transfected with vector or increasing amounts of DN Rbfox2 expression plasmids, nontreated cells were set as negative control (NC). After 48 hr incubation, the expression of Rbfox2 protein was detected by Western blotting, β-actin was detected as internal control. (E) Relative expression of Rbfox2 was normalized to β-actin in differentially-treated NRVMs. (F) The proportions of Cav1.2$_{E9*}$ channels were the values of upper band intensity divided by total intensities of upper and lower bands. n=3 independent experiments. **P<0.01, one-way ANOVA followed by a Tukey's post hoc test.

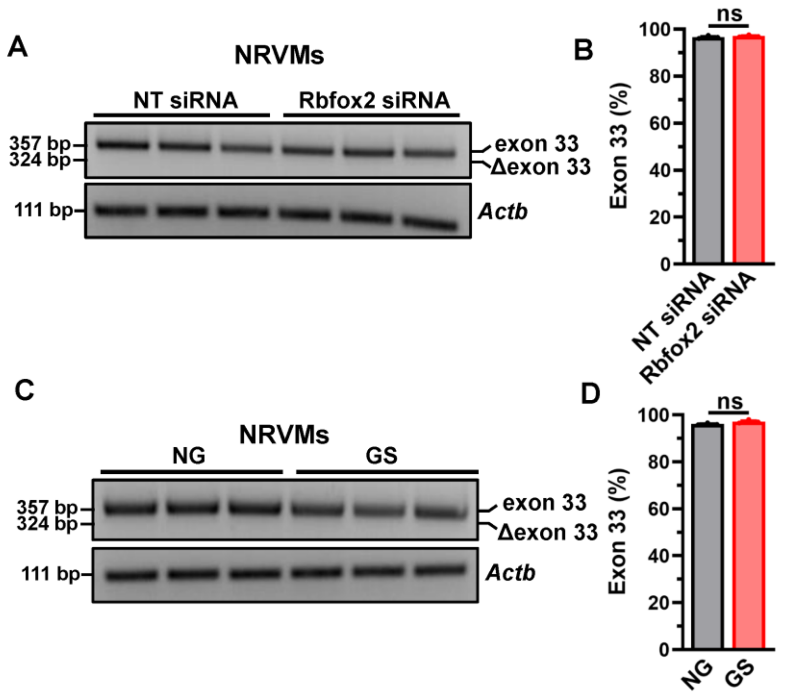

Figure S4. Application with Rbfox2 siRNA or GS does not change the proportion of Cav1.2$_{E33}$ channel in NRVMs. (A) Total RNA was extracted from NT or Rbfox2 siRNA-treated NRVMs, and PCR products of *Cacna1c* mRNA with alternative exon 33 separated on 2.5% agarose gel. *Actb* mRNA was checked as an internal reference. (B) The proportions of Ca$_V$1.2$_{E33}$ channels were the values of upper band intensity divided by total intensities of upper and lower bands. n=3 independent experiments. *P*=0.447, unpaired *t* test. (C) Total RNA was extracted from NG or GS-treated NRVMs, *Actb* mRNA was checked as an internal reference. (D) The proportions of Ca$_V$1.2$_{E33}$ channels were presented as a bar chart. n=3 independent experiments. *P*=0.1074, unpaired *t* test. ns indicates no significant differences.

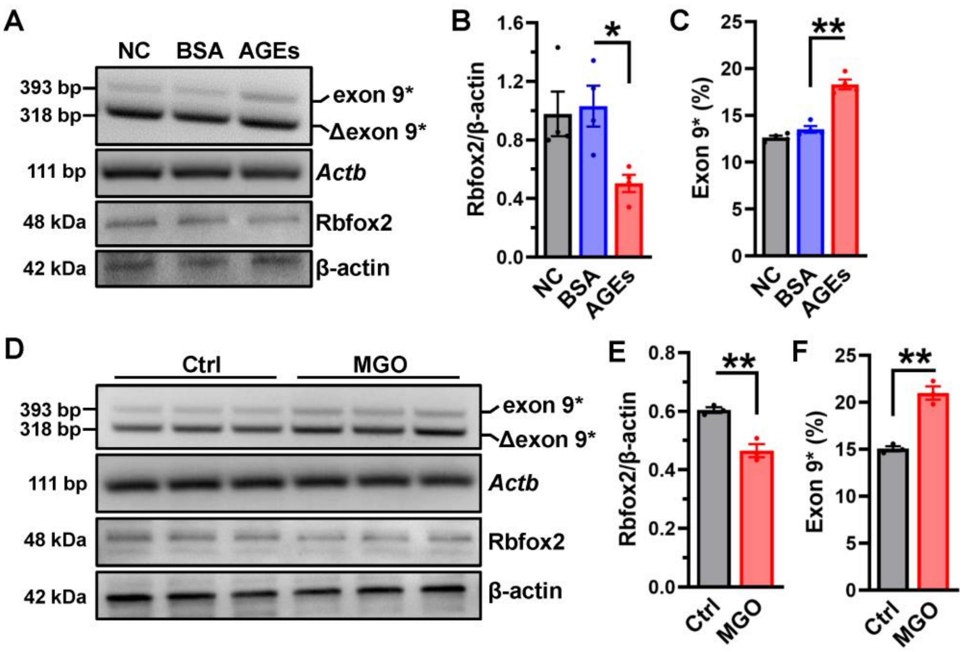

Figure S5. Treatment with AGEs or MGO decreases Rbfox2 expression but increases $Ca_V1.2_{E9*}$ channels in NRVMs. (A) Isolated NRVMs were treated with BSA or AGEs (500 μg/mL), nontreated cells were set as control. After 48 hr incubation, the endogenous expression of Rbfox2 protein was detected by Western blotting, the β-actin was detected as internal control. (B) Relative Rbfox2 expression was normalized with β-actin in differentially-treated cells. n=4 independent experiments. *P=0.0273, one-way ANOVA followed by a Tukey's post hoc test. (C) PCR products amplified from cDNA libraries of differentially-treated NRVM were separated on 2.5% agarose gel, and the values of proportion of $Ca_V1.2_{E9*}$ were analyzed. n=4 independent experiments. **P<0.0001, one-way ANOVA followed by a Tukey's post hoc test. (D) NRVMs were treated with vehicle (Ctrl) or 500 μmol/L methylglyoxal (MGO) for 48 hr, after that the cells were harvested for detecting $Ca_V1.2$ alternative exon 9* and expression of Rbfox2 by RT-PCR and Western blotting, respectively. (E) Relative expression of Rbfox2 was analyzed. n=3 independent experiments. **P=0.0045, unpaired *t* test. (F) The values for percent $Ca_V1.2_{E9*}$ channels were also presented. n=3 independent experiments. **P=0.0015, unpaired *t* test.
